# A small interfering RNA (siRNA) database for SARS-CoV-2

**DOI:** 10.1038/s41598-021-88310-8

**Published:** 2021-04-23

**Authors:** Inácio Gomes Medeiros, André Salim Khayat, Beatriz Stransky, Sidney Santos, Paulo Assumpção, Jorge Estefano Santana de Souza

**Affiliations:** 1 Bioinformatics Graduate Program, Metrópole Digital Institute, Federal University of Rio Grande do Norte, Natal, Rio Grande do Norte 59078-400 Brazil; 2 Bioinformatics Multidisciplinary Environment (BioME), Metrópole Digital Institute, Federal University of Rio Grande do Norte, Natal, Rio Grande do Norte 59078-400 Brazil; 3 Instituto do Cérebro, Federal University of Rio Grande do Norte, Natal, Rio Grande do Norte 59078-970 Brazil; 4 Biomedical Engineering Department, Center of Technology, Federal University of Rio Grande do Norte, Natal, Rio Grande do Norte 59078-970 Brazil; 5 Instituto de Ciências Biológicas, Universidade Federal do Pará, Belém, Pará 66075-110 Brazil; 6 Núcleo de Pesquisas Em Oncologia, Universidade Federal do Pará, Belém, Pará 66073-110 Brazil

**Keywords:** Databases, Data integration

## Abstract

Coronavirus disease 2019 (COVID-19) rapidly transformed into a global pandemic, for which a demand for developing antivirals capable of targeting the SARS-CoV-2 RNA genome and blocking the activity of its genes has emerged. In this work, we presented a database of SARS-CoV-2 targets for small interference RNA (siRNA) based approaches, aiming to speed the design process by providing a broad set of possible targets and siRNA sequences. The siRNAs sequences are characterized and evaluated by more than 170 features, including thermodynamic information, base context, target genes and alignment information of sequences against the human genome, and diverse SARS-CoV-2 strains, to assess possible bindings to off-target sequences. This dataset is available as a set of four tables, available in a spreadsheet and CSV (Comma-Separated Values) formats, each one corresponding to sequences of 18, 19, 20, and 21 nucleotides length, aiming to meet the diversity of technology and expertise among laboratories around the world. A metadata table (Supplementary Table S1), which describes each feature, is also provided in the aforementioned formats. We hope that this database helps to speed up the development of new target antivirals for SARS-CoV-2, contributing to a possible strategy for a faster and effective response to the COVID-19 pandemic.

## Introduction

Started in December 2019, coronavirus disease 2019 (COVID-19) rapidly transformed into a global pandemic, with an incidence of almost 100 M cases and more than 2 M deaths around the world in January 2021^1^, with a strong impact on the global economy^2^. The SARS-CoV-2 genome has a 29,903 base of single and positive-strand RNA (SARS-CoV-2 Wuhan Hu-1 strain, Accession: NC_045512), and consists of fourteen open reading frames (ORFs) which coded for twenty-seven structural and nonstructural proteins (nsps). The genome organization of SARS-CoV is similar to other CoVs and recent phylogenetic analyses indicated that SARS-CoV and the group 2 CoVs are closely related and may share a common ancestor^3^. A comparative analysis of SARS-CoV-2 and SARS-CoV showed that they present an extensive homology at genomic level, sharing approximately 79% of sequence identity^4^. Currently, there are hundreds of SARS-CoV-2 variants being sequenced^5^, a handful of vaccines have been authorized and many more vaccine candidates remain in development around the world^6^. However, despite all the scientific research and efforts, there is no specific treatment for those that were already infected by SARS-CoV-2. This scenario brought a huge demand for developing antivirals capable of targeting the SARS-CoV-2 RNA genome and RNA interference approach^7–9^ emerged as a possible solution. Small interference RNA (siRNAs) are RNA sequences about 20nt-long that, together with RNA-Induced Silencing System (RISC), bind mRNA target molecules^9,10^ inhibiting its translation and expression. Since the discovery of the RNAi mechanism in the late 90s^7^ and its effect of precisely suppressing any gene by a base sequence match, the potential of its application became evident. Soon it became a ubiquitous tool in biological research and applications, from functional genomics^11^ to biomedicine^12–15^ and pest control^16,17^. Following this 'silent revolution', in 2018 the US Food and Drug Administration approved the first RNAi therapeutic, a treatment for polyneuropathy caused by transthyretin (TTR) amyloidosis, from Alnylam Pharmaceuticals^18^.

Many studies have been proposed siRNAs development for SARS-CoV^19–21^, with reports of viral levels decrease^22^ and recent works claim that it may also work for SARS-CoV-2^23–26^. From experimental studies to patent applications, researchers have explored this approach as a potential treatment for COVID-19. Supplementary Table S3 presents a compilation of recent scientific papers, patents, and product development projects based on siRNAs with a focus on SARS-Cov-2. One of them (Gu et al^27^) performed in vitro and in vivo experiments (Syrian hamster and Rhesus macaque) with siRNA that targets RNA-dependent RNA polymerase (RdRp) gene. Two other works that developed siRNAs to target ORF-1^28^ and RdRp^29^ genes also performed, respectively, in vitro and in vivo experiments. All studies reported effective gene suppression activity of SARS-CoV-2 suggesting a promising approach for treating COVID-19. Furthermore, as occurred during the SARS-CoV epidemic in 2003^30,31^, many patent applications for SARS-CoV-2^32^ have been filed. (see *Patents submissions* at Supplementary Table 3). A development project for clinical application includes a multimillionaire endeavor led by Vir and Alnylam® Pharmaceuticals^33^ companies to develop an RNAi therapeutic (called VIR-2703) for COVID-19 (see *Related projects* at Supplementary Table S3).

A critical step in the development of RNAi-based therapies is the design of siRNAs. To find potential regions in diverse coronaviruses with matches to SARS-CoV-2, identifying many of them in SARS-CoV, the closest homolog, researchers^34^ used Immune Epitope Database and Analysis Resource (IEDB)^34^. Chen et al^35^ applied a window of 3000 nucleotides with a step of 1500 over the reference SARS-COV-2 genome seeking 1–25nt regions called 'free segments'. Besides, siRNAs databases targeting a broad range of viruses^36–38^ have been developed. Recently, researchers developed a SARS-CoV-2 oligonucleotide sequence database, to improve the SARS-CoV-2 detection and treatment methods, providing sequences with the lowest and highest conservation levels^39^.

In this work, we presented a SARS-CoV-2 targets database to support the development of siRNA approaches and speed up RNAi design, by providing a set of possible targets and siRNA sequences with the required information for choosing the most appropriate targets for new siRNAs. Unlikely cited databases, which are manually curated and provide only a small set of siRNAs chosen for specific targets (see *siRNA computational identification and design papers* at Supplementary Table S3), we apply a sliding-window approach for covering whole SARS-CoV-2 genomic space, extracting every possible siRNA sequence of 18, 19, 20, and 21 nucleotides. This methodology generated a comprehensive database that enables researchers to assess solutions capable of targeting any region of the virus but also to select homologous regions between the circulating variants. It also enabled 100% of matches with siRNAs published by similar works (see column *% of siRNAs present in the proposed database* at Table S3). The database presents more than 170 features, including thermodynamic information, base context, target genes, and alignment information against diverse SARS-CoV-2 strains, together with scores and predictions collected from three siRNA efficiency prediction tools. It is worth mentioning that the various laboratories around the world have distinct expertise and goals for siRNAs development, therefore, all this coordinated information will enable users to select, with higher confidence, targets that best match a broad set of conditions for designing even more efficient siRNAs.

## Results

### Database analysis and statistics

**Figure 1:**
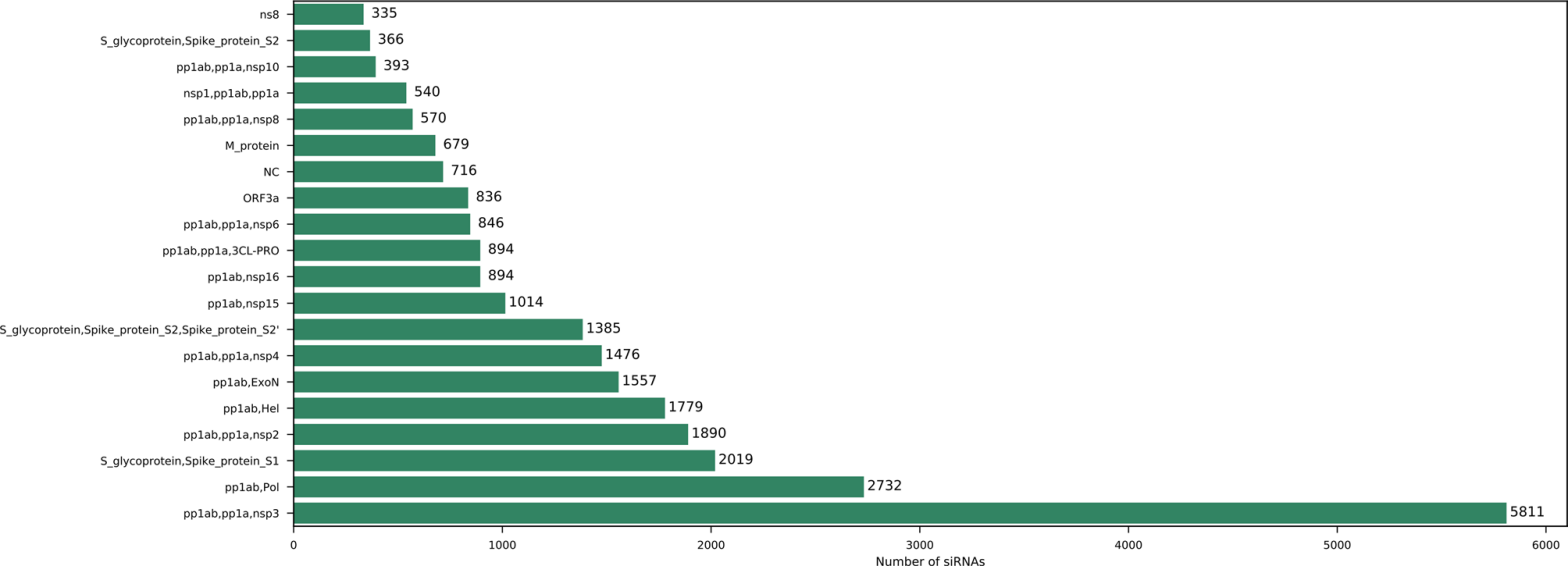
Distribution of 21nt siRNAs across the twenty more 21nt-siRNA-abundant genes from SARS-CoV-2. Number of siRNA targets per gene, displayed in horizontal bars. Overlapping genes are displayed at the same line, separated by comma.

The proposed database displays a total of 119,526 siRNAs divided into four different sizes, ranging from 18 to 21 nucleotides (see Table 1 for the number of siRNAs of each length), and covering more than 170 features (Supplementary Table S1 describes each feature). The column *Annot*, for example, indicates which gene (or genes) a siRNA can target, and should be consulted if the user wants to design siRNAs focused on inhibiting the activity of a single gene or a group of overlapping genes. Figure 1 provides the distribution of 21nt siRNAs across the twenty-most siRNA-abundant SARS-CoV-2 genes. It can be noticed that gene overlapping *pp1ab,pp1a,nsp3* comprises about 20% of all siRNAs (5811), more than the double of the next most siRNA-abundant gene, the gene overlapping *pp1ab, Pol*, which can be targeted by 2732 siRNAs (about 9% of all 21nt ones). Otherwise, gene overlapping *S_glycoprotein, Spike_protein_S2* and *nsp8* holds the lowest number of siRNAs: 366 and 335, respectively (about 1% each).

**Table 1:** Number of siRNAs of each length.

Length	Number of siRNAs
18	29,883
19	29,882
20	29,881
21	29,880
Total	119,526

**Figure 2:**
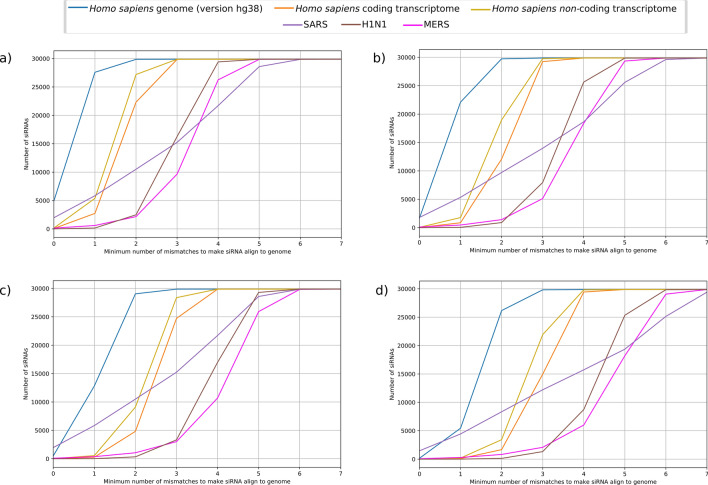
Number of mismatches against Human genome and transcriptomes. The number of antisense (**a**) 18nt-long, (**b**) 19nt-long, (**c**) 20nt-long, and (**d**) 21nt-long siRNAs with different mismatches against Human genome, Human coding and non-coding transcriptome, and MERS, SARS, and H1N1 genomes.

We also aligned all siRNAs to the Human genome, Human coding and non-coding transcriptomes, SARS, MERS, and H1N1 genomes, with Bowtie version 1.1.0, to identify if siRNAs could off-target regions in those organisms, thus presenting cross-reactivity with them. According to Yamada et al.^40^, a minimum of three mismatches against the human genome is necessary to guarantee that the siRNA will not anneal off-target regions, hence increasing its effectiveness. Figure 2a–d illustrate the growth of siRNAs quantities as the minimum number of necessary mismatches to have alignment increases. The results show that virtually all 18nt siRNAs can match the human genome and transcriptomes (coding and non-coding) with three mismatches. This number, however, increases to four mismatches considering 19nt and 20nt (Fig. 2b, c), and to five considering the 21nt length (Fig. 2d). It can be observed that in each length, about 2500 siRNAs match perfectly with some region of SARS. Regarding MERS and H1N1, about 2500 18nt siRNAs can match regions of those viruses, when the minimum number of allowed mismatches is two (Fig. 2a). This number is overcome by 19–21nt siRNAs only when the number of mismatches is increased to three (Figs. 2b–d). Finally, it is also important to note that while all 18nt and 20nt siRNAs match some regions from MERS, SARS, and H1N1 using at least six mismatches, the number of mismatches increases to seven for 19nt and 21nt siRNAs.

To analyze the siRNAs’ effectivity to address strains sets from different populations, other alignments with Bowtie version 1.1.0 were performed, this time with siRNAs against SARS-CoV-2 strains available at the Global Initiative of Sharing All Influenza Data (GISAID) database (https://www.gisaid.org/), coming from nine countries (see Methods). This analysis indicate, for example, which siRNAs are more suitable for a specific country, given its matches with the strains. The columns BV to CD, CN to CV, and DF to DN from database spreadsheet files (see Supplementary Table S1) provide the number of genomes from each strains country set that *natural sense*, *synthetic sense*, and *antisense* respectively have a perfect match with (the total number of strains genomes is available at columns’ headers).

**Figure 3:**
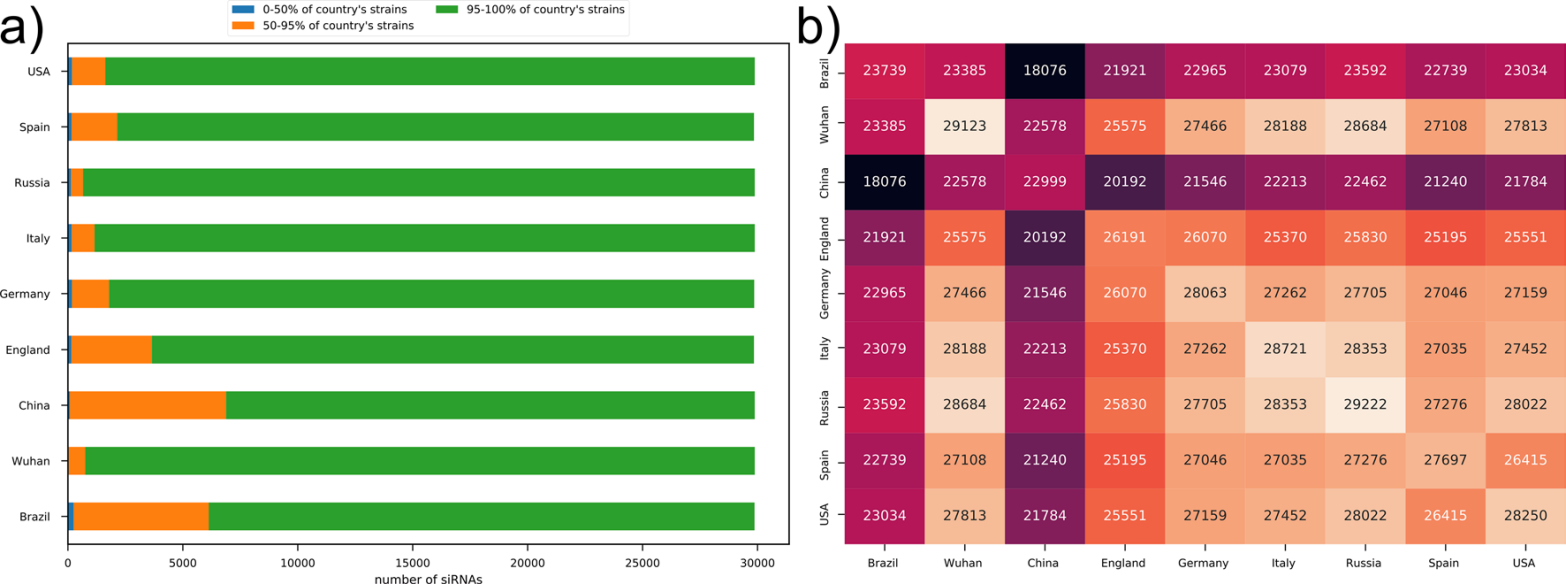
Coverage of 21nt siRNAs across strains from nine countries. (**a**) Targeting coverage of 21nt siRNAs across nine countries, divided in three layers: (1) 0–50% of the country’s strains; (2) 50–95% of the country’s strains; (3) 95–100% of the country’s strains. (**b**) Intersection matrix displaying the number of siRNAs with coverage higher than 95% that each country pair shares.

Figure 3a yields a big picture of country coverage for each 21nt *antisense* sequence siRNA. The majority of siRNAs encloses more than 95% of the strains from all countries. In spite of that, a lesser but significant portion of siRNAs encloses between 50 and 95% of the strains from all countries, with China and Brazil presenting the highest numbers (6796 and 5881 siRNAs, respectively). Russia has the smallest portion of such siRNAs (524 siRNAs). Figure 3b displays an intersection matrix from siRNAs that enclose more than 95% of the strains from each country, providing the number of siRNAs that have a match simultaneously with each country pair. Brazil and China produce similar profiles: while the former shares between 18.000 and 24.000 siRNAs with more than 95% of country coverage with other countries, China shares between 18.000 and 23.000 siRNAs. On the other hand, England’s pairs vary between 20.000 and 26.500 siRNAs, and it is possible to see a “cluster” formed between Germany, Italy, Russia, Spain, and USA, wherein each pair shares more than 27.000 siRNAs. These results suggest that many of the 21nt siRNAs in our proposed database have potential to be used worldwide, given such sharing power.

Supplementary Table S2 shows the number of 21nt siRNAs with more than 95% of coverage against the variants presented in each country. It can be noticed that same number of siRNAs (570) against the overlapping genes *pp1ab, pp1a, nsp8* is observed across all countries but China (where it has 410), and the overlapping genes *pp1ab, pp1a, 3CL-PRO*, across all countries (with 894 siRNAs in each one) except China (828 siRNAs) and Russia (873). Otherwise, the overlapping genes *pp1ab, pp1a, nsp10* present the same number of siRNAs (393) in all countries but China (358) and Spain (334). Although Russia and China do not share the same number of siRNAs in any gene, Russia does with Wuhan in the overlappings *pp1ab, ExoN* (1557), *pp1ab, nsp16* (894), and *pp1ab, pp1a, nsp10* (393 siRNAs). Besides, the number of siRNAs with more than 95% of coverage in Russia that target *S_glycoprotein* is the same number of at least one other country. These results indicate that it is possible, with the proposed database, to screen for siRNAs with effectivity directed to a specific gene (or group of overlapping genes) with either a potential global application or to a specific set of populations of interest.

The possible toxicity of a siRNA in humans is an important aspect that must be taken into account during design processes, and can be handled at diverse levels, from the molecular one to the issue of having off-targeting capabilities (as previously mentioned and discussed). Regarding sequence level, where the proposed database is located, a set of proposals can be found in the literature^40,41^ related to how to assess the toxicity of a siRNA. Seeking to evaluate the toxicity level of 21nt siRNAs from the proposed database, to check its ability to handle these issues, we applied a filtering set of four masks based on previous works^40,41^ (see Table 2, section Toxicity). A total of 26,629 siRNAs (about 89% of the 21nt database) were considered toxic, and 3251 atoxic (about 11%). Country coverage of atoxic siRNAs (Supplementary Figure S1a,b) follows the same visual pattern from Fig. 3, this time with pairs involving Brazil or China each one varying from 1,800 to 2,500 siRNAs; England, from 2,000 to 2,900, and the “cluster” formed between Germany, Italy, Russia, Spain, and USA, with more than 2,800 siRNAs. Their distribution across Top 20 siRNA-abundant SARS-CoV-2 genes (Supplementary Figure S2) repeats the pattern of overlappings *pp1ab, pp1a, nsp3* and *pp1ab, Pol* with the highest quantities (436 and 330, respectively), and now *nsp8* gene is the third least, covering 64 siRNAs (overlappings *pp1ab, nsp16*, and *pp1ab, pp1a, nsp9* fill the list with 62 and 59 siRNAs, respectively).

**Table 2:** Example of siRNAs filtering criteria according to diverse characteristics.

Characteristic	Database feature name(s)	Database spreadsheet column(s)	Filter^reference^
Toxicity	hs	CY	Has at least three mismatches with Human genome^40^
	hs_cds	CZ	Has at least three mismatches with Human coding transcriptome^40^
	hc_ncrna	DA	Has at least three mismatches with Human non-coding transcriptome^40^
	UUUU and GCCA	BC and BD	Does not have neither UUUU neither GCCA tags in its sequence^41^
Effectivity	sGG, sCC, fAA, fTT	R, S, V, and U	Has G or C at 5′ of antisense strand and A or T at 5′ of sense strand^43^
	GC	BA	Has GC content between 36 and 52%^44^
	Palindromic_AS	BL	Does not have palindromic subsequences^46^
	Hairpin	DX and EV	Does not make hairpin
	SelfAnnealing	DY and EW	Does not self-anneals
Populations and genomes coverage	Brazil (57), Wuhan (48), China (41), England (3416), Germany (180), Italy (82), Russia (154), Spain (410), USA (4725)	DF to DN	Matches with as many as possible SARS-CoV-2 from different countries
	mers, sars, h1n1	DB, DC, DD	Matches with MERS, SARS and H1N1 genomes
Stability	DG	HO	Structural stability lies between -32 to -28 kcal/mol^45^
	Tm	EO	Melting temperature is around 20ºC^42^
	DDG	HP	Terminal duplex asymmetry (ΔΔG) equal or higher than 2 kcal/mol^45^
Effectiveness prediction by predictors	Predicted Eficacy	FY	Effectiviness prediction by ThermoComposition21
	GOOD	GJ	Effectiviness prediction by SSD
	GOOD	HN	Effectiviness prediction by si-shRNA selector

Criteria filtering sets from literature regarding (a) Toxicity, (b) Efficiency, (c) Populations and genomes coverages, (d) Stability, and (e) Effectiveness prediction. First column indicates each such group of characteristics; second column, the name of database feature whose filtering criteria (last column) is related; third column, the spreadsheet database column related to database feature name; and last column, the filtering criteria.

**Figure 4:**
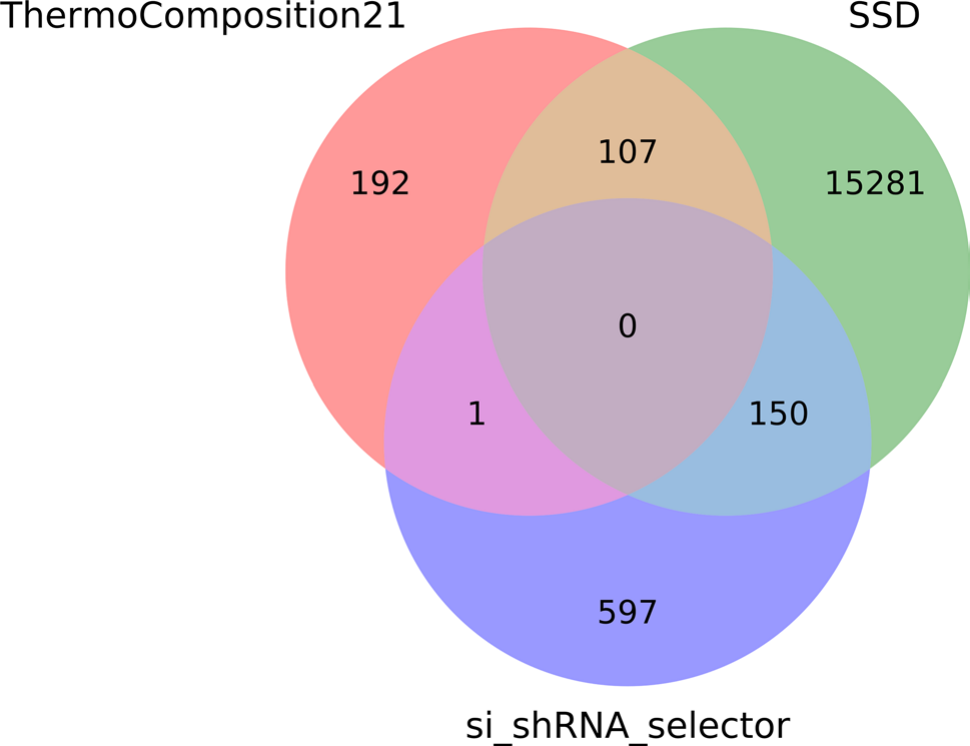
Venn diagram of three predictors for 21nt-long antisense sequences classified as efficient. The number of siRNAs that are simultaneously considered as effective by each pair of tools from ThermoComposition21, SSD and si_shRNA_selector.

As stated in Methods, we applied over all the siRNAs three efficiency prediction tools to assess their inhibition power. Figure 4 illustrates the number of 21nt *antisense* siRNA sequences predicted as effective by every single predictor, and the quantities predicted by more than one. It can be seen that no siRNA was unanimously considered effective, while approximately 53% of them (15,821 siRNAs) were considered as such just by SSD. Besides, a single siRNA was predicted as effective by both ThermoComposition21 and si_shRNA_selector.

The literature^40–46^ regarding siRNA effectiveness indicates that three main characteristics should be considered during the design processes: Toxicity, Stability, and Effectivity. We examined whether these criteria could be assessed using the variables of our proposed database. Table 2 addresses the mapping between the features of the database and literature filtering criteria. It is possible to see that there is at least one available feature for each criterium. For example, the Toxicity filter that has *neither UUUU nor GCCA tags in its sequence,* matched with database columns *UUUU* and *GCCA*, which tells the user exactly whether a siRNA of interest has such tags. Database column *GC*, on the other hand, gives siRNA GC% content information, so it can be used to evaluate Effectivity criterion as *GC content between 36 and 52%*. Thus, the proposed database is suitable for effective siRNAs selection under diverse and customized user requirements.

### Database use and access

The proposed database is distributed as a set of five files, available in spreadsheet and CSV formats. One of them is a metadata table, containing the description of each column (Supplementary Table S1 replicates such table), and the remaining ones correspond to *target region* sequences of a specific length. Here we will present how a researcher can use this database with an illustrative example. Suppose a user wants to select siRNAs with 21 nucleotides length. In this case, the user will access either the file *21bases.xlsx* or the file *21bases.csv* (for purposes of this example, it will be considered that the user has accessed *21bases.xlsx*). After opening it in a spreadsheets editor, the next step is selecting siRNAs whose properties match the user requirements. Assume that the user wants a siRNA that has little or no homology with the human genome, can act over as much as possible British SARS-CoV-2 strains, and its first dinucleotide is AA. This last requirement is achievable by applying a filter over column *sAA* (see Supplementary Text S1 and Supplementary Table S1) to show only lines with value 1 on it (see Supplementary Text S1), decreasing the number of siRNA candidates from 29,880 to 2858. For little or no homology with the human genome, the number of mismatches against human sequences must be at least three^40^. Filtering table to display lines with at least a value of three at spreadsheet columns BO, BP, BQ, CG, CH, CI, CY, CZ, and DA (Supplementary Table S1) now reduces candidates from 2858 to 999. Finally, to approach as many British strains as possible, spreadsheet column *England (3012)* (Supplementary Table S1) can be filtered to display only the three highest values, for example, which reduces candidates from 999 to 10 candidates. Such a reduction not only saves wet-lab test costs but also ensures that selected siRNAs meet the main user requirements.

## Discussion

Small interfering RNAs (siRNAs) are double-stranded non-coding RNA molecules of 18–25 base pairs long, which regulate the expression of genes by a phenomenon known as RNA interference (RNAi). Although their therapeutic use had imposed many challenges to overcome limited effectiveness and potential toxicity effects in the early applications^12,47^, several siRNAs have been developed as potential therapies against viral infections with limited treatment options and accessible target cells, like hepatitis B virus^48^, Ebola^49^, and respiratory syncytial virus^50^. For SARS-CoV, the effect of prophylactic and therapeutic activities of siRNAs in Rhesus monkeys (*Macaca mulatta*) was evaluated in Li et al^22^. The researchers used two duplex siRNAs, targeting the SARS-CoV genome in the spike and NSP12 protein-coding regions. They found that siRNAs provide relief of fever caused by SARS-CoV infection, reducing the viral load and decreasing acute diffuse alveolar damage. In addition to proving the effectiveness of these siRNAs in prophylactic and therapeutic activity, the experiments did not show any signs of toxicity related to the use of siRNAs as therapy.

Regarding SARS-CoV-2, three different in vitro and in vivo studies^27–29^ successfully reported siRNAs application as a potential treatment for COVID-19 (see Supplementary Table S3, *Pre-clinical or human-clinical test studies* line). Furthermore, researchers have applied for a large number of patents related to vaccines or drug development reported siRNAs targeting M, N, and E protein genes, PI4KB, RdRp, ORF3a gene, among others (reviewed in^30–32^). As expected, many of these and new studies have been reversed in patent applications applied to COVID-19. Two patents reported a preparation method of a CoViD-19 antisense RNA multivalent vaccine (CN111330003A) and a dsRNA vaccine (CN111321142A), targeting ORF1ab, 3′ UTR, and S, E, M, or N genome region. For the development of biological medicines aiming to prevent and/or treat Covid-19, siRNAs were developed for conserved regions of the SARS-CoV-2 (CN111139241A and CN111139242A). According to the authors, siRNA modified by the invention has an obvious inhibition effect on the gene, with a great clinical significance for treating COVID-19 pneumonia. Also applying siRNA molecules, other patents report the suppression of SARS-CoV-2 replication, by targeting the ORF1 (ORF1a, ORF1b) and N genes (RU2733361C1) or described an effective inhibition of the expression of virus key protein by targeting the gene sequence of RDRP enzyme or S protein (CN111518809A). Meanwhile, biotechnology companies have invested heavily to consolidate RNAi therapeutics for COVID-19. A collaborative team of Alnylam Pharmaceuticals and Vir Biotechnology developed an aerosolized delivery of siRNAs optimized for lung uptake, and are conducting in vitro and in vivo tests, whereas Sirnaomics perform preclinical studies with a respiratory-specific siRNA formulation that is delivered by a customized handheld nebulizer device^51^. Based on these studies and evidence, we believe that siRNA-based therapies are a promising tool for fighting epidemics. Furthermore, the siRNAs experimentally validated in above papers and also presented in our bank indicate that the proposed database can effectively help to achieve this goal.

Despite the exciting results obtained by this technique, researchers still face many challenges, and one of the most critical is the avoidance of nonspecific toxicity in therapeutic applications. According to Setten et al.^12^, the main sources of toxicity that have considerably affected clinical RNAi drug development are related to (1) Immunogenic reactions to dsRNA, (2) Toxicity of excipients, (3) Unintended RNAi activity, and (4) On-target RNAi activity in non-target tissues. Some of these problems are largely mitigated by the development of excipients limited to a small number of chemical components that are individually verified for low toxicity, or by choosing specific delivery routes, like lipidic nanoparticles complexes and other non-viral vectors^52,53^. Although the sequence features from siRNAs are insufficient to evaluate the assertion of delivery at the intended target area, it is an essential information to evaluate efficiency and possible toxic effects^43,44^. In this paper we worked on specific parameters based on siRNAs sequence features, that evaluate molecule stability and its potential to interact with off-target regions and pathways from human coding and non-coding transcriptome. Careful evaluation of these parameters will help to optimize the design and effective development of SiRNAs for each given objective.

The design of siRNAs is a challenging procedure because sometimes minor changes in its nucleotide sequence can alter its functionality^42^. As reported by Alagia et al^54^, specificity, potency, and efficacy of siRNA-mediated gene silencing can be determined by analyzing the siRNA nucleotide sequence, hence its inability to bind to unintended regions (off-targets) is an important factor that must be strongly taken into consideration. Therefore, we proposed a SARS-CoV-2 targeted siRNAs database with sequence and thermodynamic stability information, to help the evaluation of important factors related to their efficacy and optimize the decision process towards choosing the best ones as target antiviral solutions. Considering that each laboratory has its own technology context and expertise in designing siRNAs of specific lengths, we provide a list of siRNAs varying from 18 to 21 nucleotides-length, aiming to meet the range of possible lengths used in the design process.

The analysis of 21nt siRNAs showed the overlapping genes with most siRNAs (5811, 20% of the total number) involve *pp1ab*. Once this gene covers about 70% of the virus genome (21287nt)^3^, it is natural that most of the siRNAs fall in it. Thus, the database gives the option to screen either for siRNAs with higher or lesser gene-specificity, in which the higher the number of overlapping genes that a siRNA can target, the higher the chances are to it be more effective because a larger set of viral functions will be compromised. On the other hand, gene *nsp8* covers only about 2% of the genome (594nt)^3^, which may explain the reduced number of siRNAs that target it. The gene *nsp3*, which is present in the most target *pp1ab*, participates in the process of viral transcription and replication^55,56^. Since gene distribution analysis of siRNAs considered atoxic (see Results and Figure S2) revealed the same distribution pattern from the whole dataset, it can be suggested that *nsp3* is a good target candidate for siRNAs design and development, given its abundance and function.

An early 2020 variation analysis study^57^ over SARS-CoV-2 strains from diverse countries reported homology levels between 99 and 100% for all strains. These countries presented the highest numbers of siRNA sharing pairs (Fig. 3), thus supporting the idea of high conservation areas in the SARS-CoV-2 genome. This can also resemble in Supplementary Table S2, where some genes have the same number of siRNAs that are capable to target at least 95% of strains from diverse countries. These results indicate that, although siRNAs from the proposed database can not target mutation sites from new SARS-CoV-2 strains, the fields ranging from BV to CD spreadsheet columns (see Supplementary Table S1) help to identify homology regions common to all strains from a specific country.

Numerous works have been proposing methods and guidelines for choosing the best siRNAs by analyzing their sequence characteristics^43,44,58,59^, for which two broad reviews are available^42,60^ (some of them are briefly discussed at Supplementary Text S1). Given the importance of such guidelines and also the characteristics involved in their formulation, we decided to insert all this information into the database, so that users can select their best siRNAs from instructions already published, or by drafting their own rules from their expertise and specific objective. In this way, all the information contained in the database can be used in a customized and cost-effective manner. For example, our proposed database provides information regarding the bases, GC, and AU context, so as the quantities of each RNA nitrogenated base in sequences, besides information about the presence of UUUU and GCCA, considered toxic motifs^41^, so any user with a proper efficacy evaluation method (or anyone provided by literature) can easily evaluate siRNAs with this database at disposal. It also provides thermodynamic information collected from the application of three predictors^45,61,62^, thus enabling users to have a deeper look at siRNAs’ properties, and choose the best ones according to their specificities. As it can be seen in Fig. 4, they have high divergence when setting a siRNA as efficient or not, which suggests that they must be used in a complementary way. Due to the genetic diversity and variability of SARS-CoV-2^63^, a siRNA that is highly efficient over one strain may not be when applied to another. Hence, we also provide information about similarity within strains from diverse countries, such that users will benefit from the opportunity of input geographical specificity and even more customization to their decision process.

Ensuring that siRNAs are not capable of targeting human sequences (off-targets) is also another important requirement, for which a minimum of three mismatches is necessary to meet it^40^. Thus, similarity information with the human genome, coding, and non-coding transcriptome, is also available in our database. As it was shown in the Database Analysis & Statistics session, virtually all 18nt-long siRNAs matched with such genome and transcriptomes with at least three mismatches, corroborating with the literature statement^40^. To the best of our knowledge, this is the first database to figure SARS-CoV-2 siRNAs similarity information against human coding and non-coding transcriptomes, giving to users even more confidence power over siRNAs specificity. To investigate how it is possible to use the database for customized efficient siRNAs selection, we have elicited from literature filtering criteria regarding siRNA activity, such as Toxicity, Stability, and Efficiency. Table 2 showed that all the listed criteria can be handled using database features, which allows users to delimit thresholds and look at features that maximize desired skills that a siRNA must have to fulfill their requirements, to specialize the search space for their needs. It is important to note, however, that this database has not its use limited to biomedical applications: users can exploit their features to other biotechnological applications that have distinct requirements and explore database information in different ways. This is a clear advantage of our database over others already developed—the fact the whole possible siRNAs set are available opens the potential for groups with diverse specialties to work with them for different applications beyond healthcare ones.

In this work, we presented a database to support the development of new target antivirals for SARS-CoV-2 using RNAi technology. We hope that the development of new antiviral products can not only be facilitated and accelerated but that the presented database helps to generate even more efficient solutions to silence the virus, contributing to the control of the pandemic. Given the urgency to provide this information for the scientific community, we made available the database as a set of tables files in spreadsheet and CSV formats, however, a webpage for more user-friendly and interactive access to the data will be released soon. Finally, it is important to stress that the approach presented here can be successfully applied for exploring the genomic information of other viruses, including the ones that may represent a threat to new pandemic events.

## Methods

Although siRNAs length can vary from 18 to 25 nucleotides^64^, synthetic ones should range from 19 to 21nt^65^, according to ThermoFisher siRNA Design Guidelines^66^. Thus, the proposed database provides information about each possible 18–21 nucleotides siRNA target region from SARS-CoV-2, one table for each length. Moreover, tools employed for assessing siRNAs efficiency^45,61,62^ operate over sequences lying in that range, which reinforces our choice. Since they present the same columns, we explain here the development process only for the 21-length table.

SARS-CoV-2 reference genome was collected from NCBI (code NC_045512) and a sliding window of 21nt-long and step 1 (this parameter is used in all tables, independently of length) were used to traverse the genome. Table 1 indicates the total number of sequences obtained for each length. Seven new sequences sets were then generated from the obtained sequences set (called *target region*), following the aforementioned ThermoFisher guidelines, and suggestions from our collaborators: (1) *natural sense*, by removing the first 5′-end dinucleotide from *target region* sequences; (2) *oligo natural sense,* replacing thymine with uracil over *natural sense* set; (3) *synthetic sense,* by replacing first 3′-end dinucleotide from *natural sense* sequences with TT; (4) *oligo synthetic sense*, by replacing of thymine with uracil over *synthetic sense* sequences; (5) *antisense*, from the reverse complement of *target region*; (6) *oligo antisense*, by replacing thymine with uracil over *antisense* sequences; and (7) *oligo antisense rev* set, by reversing *antisense* sequences.

*Natural sense* sequences were then aligned against (a) SARS-CoV-2 reference genome, to verify which genes they align with; (b) the human genome (NCBI accession code GRCh37) and coding and non-coding transcriptome, to verify potential cross-reaction with off-target transcripts; (c) SARS-CoV-2 strains available at Global Initiative of Sharing All Influenza Data (GISAID) database (https://www.gisaid.org/) coming from Brazil, China (Wuhan region only and whole country less Wuhan), England, Germany, Italy, Russia, Spain and USA; and (d) reference genomes of MERS virus (NCBI MG987420), SARS-CoV (NCBI NC_004718) and Influenza virus genome (NCBI NC_026438), aiming to assess whether siRNAs are capable to target regions from those viruses and strains. Bowtie^67^ version 1.1.0 was used as the aligner, using the following flags: -a, -S, --pairtries equals to 4, -p equals to 40, -n equals to 3, -l equals to 7 and -f. Flag -e was used, being equals to 150 when aligning against the human genome, 10 for GISAID strains, and 220 for remaining genomes. Sequence properties regarding base context and alignment information were calculated from the above sequences sets and performed alignments (see Supplementary Text S1). Thermodynamic information and expected efficiency of candidates siRNA designed for targeting those regions was calculated with OligoCalc^68^ and three predictors, namely ThermoComposition21^61^, SSD^62^, and si-shRNA Selector^45^. Finally, we elicited criteria filtering sets from literature regarding (a) Toxicity, (b) Efficiency, (c) Populations and genomes coverages, (d) Stability, and (e) Effectiveness prediction, which are summarized at Table 2, and then analyzed whether they could be assessed with the features of proposed database.

## Supplementary Information

Supplementary Information

## Data Availability

The spreadsheet and CSV files regarding database and metadata tables are available in a zip-compressed file at Open Science Framework (https://doi.org/10.17605/OSF.IO/WD9MR) and mirrored at http://www.bioinformatics-brazil.org/siRNAdb/sirnas_cov_db.zip. Codes and binaries regarding software employed to build the database are available at https://github.com/inaciomdrs/sirna_db_building_protocol. A protocol describing technical details about database generation is currently available at Nature Protocol Exchange (https://doi.org/10.21203/rs.3.pex-1207/v1). A preprint version of this paper is available at bioRxiv (https://doi.org/10.1101/2020.09.30.321596).

## References

[1] World Health Organization. Coronavirus disease (COVID-19): Situation report, 198. (2020).

[2] Tu Y-F (2020). A review of SARS-CoV-2 and the ongoing clinical trials. Int. J. Mol. Sci..

[3] Wu A (2020). Genome composition and divergence of the novel coronavirus (2019-nCoV) originating in China. Cell Host Microbe.

[4] Zhou P (2020). A pneumonia outbreak associated with a new coronavirus of probable bat origin. Nature.

[5] Cyranoski D (2021). Alarming COVID variants show vital role of genomic surveillance. Nature.

[6] COVID-19 vaccine tracker. https://www.raps.org/news-and-articles/news-articles/2020/3/covid-19-vaccine-tracker. (2021).

[7] Fire A (1998). Potent and specific genetic interference by double-stranded RNA in Caenorhabditis elegans. Nature.

[8] Lee RC, Feinbaum RL, Ambros VT (1993). The C. elegans heterochronic gene lin-4 encodes small RNAs with antisense complementarity to lin-14. Cell.

[9] Qureshi A, Tantray VG, Kirmani AR, Ahangar AG (2018). A review on current status of antiviral siRNA. Rev. Med. Virol..

[10] Spurgers KB, Sharkey CM, Warfield KL, Bavari S (2008). Oligonucleotide antiviral therapeutics: Antisense and RNA interference for highly pathogenic RNA viruses. Antiviral Res..

[11] Xia H, Mao Q, Paulson HL, Davidson BL (2002). siRNA-mediated gene silencing in vitro and in vivo. Nat. Biotechnol..

[12] Setten RL, Rossi JJ, Han S-P (2019). The current state and future directions of RNAi-based therapeutics. Nat. Rev. Drug Discov..

[13] Wang S-T (2011). RNA interference-mediated silencing of Foxo3 in antigen-presenting cells as a strategy for the enhancement of DNA vaccine potency. Gene Ther..

[14] Kabekkodu SP (2020). Cluster miRNAs and cancer: Diagnostic, prognostic and therapeutic opportunities. Wiley Interdiscip. Rev. RNA.

[15] Poller W (2018). Non-coding RNAs in cardiovascular diseases: diagnostic and therapeutic perspectives. Eur. Heart J..

[16] Sherman JH (2015). RNAi technologies in agricultural biotechnology: The toxicology Forum 40th annual summer meeting. Regul. Toxicol. Pharmacol..

[17] Fletcher SJ, Reeves PT, Hoang BT, Mitter N (2020). A Perspective on RNAi-based biopesticides. Front. Plant Sci..

[18] Garber K (2018). Alnylam launches era of RNAi drugs. Nat. Biotechnol..

[19] Shi Y (2005). Inhibition of genes expression of SARS coronavirus by synthetic small interfering RNAs. Cell Res..

[20] Meng B, Lui Y-W, Meng S, Cao C, Hu Y (2006). Identification of effective siRNA blocking the expression of SARS viral envelope E and RDRP genes. Mol. Biotechnol..

[21] Wang Z (2004). Inhibition of severe acute respiratory syndrome virus replication by small interfering RNAs in mammalian cells. J. Virol..

[22] Li B-J (2005). Using siRNA in prophylactic and therapeutic regimens against SARS coronavirus in Rhesus macaque. Nat. Med..

[23] Ghosh S, Firdous SM, Nath A (2020). siRNA could be a potential therapy for COVID-19. EXCLI J..

[24] Liu C (2020). Research and development on therapeutic agents and vaccines for COVID-19 and related human coronavirus diseases. ACS Cent. Sci..

[25] Uludağ H, Parent K, Aliabadi HM, Haddadi A (2020). Prospects for RNAi therapy of COVID-19. Front. Bioeng. Biotechnol..

[26] Lundstrom K (2020). Coronavirus pandemic—Therapy and vaccines. Biomedicines.

[27] Gu SH (2020). A Small interfering RNA lead targeting RNA-dependent RNA-polymerase effectively inhibit the SARS-CoV-2 infection in Golden Syrian hamster and Rhesus macaque. bioRxiv.

[28] Ambike S (2020). Systematic analysis of RNAi-accessible SARS-CoV-2 replication steps identifies ORF1 as promising target. Res. Square.

[29] Khaitov, M., Nikonova, A., Shilovskiy, I. & Kozhikhova, K. Silencing of SARS-CoV-2 with modified siRNA-peptide dendrimer formulation. *Authorea* (2021). 10.1111/all.14850 PMC8251148 33837568

[30] Kumar V, Jung Y-S, Liang P-H (2013). Anti-SARS coronavirus agents: A patent review (2008–present). Expert Opin. Ther. Pat..

[31] Nascimento Junior JAC (2020). SARS, MERS and SARS-CoV-2 (COVID-19) treatment: a patent review. Expert Opin. Ther. Pat..

[32] Zhou W, Chen D (2021). Emerging patent landscape for gene therapy as a potential cure for COVID-19. Math. Probl. Eng..

[33] Reuters Staff. Vir, Alnylam plan human trials by year-end for potential COVID-19 therapy. *Reuters* https://www.reuters.com/article/us-health-coronavirus-vir-biotech-idUKKBN22G1H1. (2020).

[34] Grifoni A (2020). A sequence homology and bioinformatic approach can predict candidate targets for immune responses to SARS-CoV-2. Cell Host Microbe.

[35] Chen W, Feng P, Liu K, Wu M, Lin H (2020). Computational identification of small interfering RNA targets in SARS-CoV-2. Virol. Sin..

[36] Thakur N, Qureshi A, Kumar M (2012). VIRsiRNAdb: a curated database of experimentally validated viral siRNA/shRNA. Nucleic Acids Res..

[37] Gupta, N., Zahra, S., Singh, A. & Kumar, S. PVsiRNAdb: A database for plant exclusive virus-derived small interfering RNAs. *Database* **2018**, (2018). 10.1093/database/bay105 PMC6181178 30307523

[38] Tyagi A (2011). HIVsirDB: A database of HIV inhibiting siRNAs. PLoS ONE.

[39] Carneiro J, Gomes C, Couto C, Pereira F (2020). CoV2ID: Detection and therapeutics oligo database for SARS-CoV-2. bioRxiv.

[40] Yamada T, Morishita S (2005). Accelerated off-target search algorithm for siRNA. Bioinformatics.

[41] Fedorov Y (2006). Off-target effects by siRNA can induce toxic phenotype. RNA.

[42] Fakhr E, Zare F, Teimoori-Toolabi L (2016). Precise and efficient siRNA design: A key point in competent gene silencing. Cancer Gene Ther..

[43] Ui-Tei K (2004). Guidelines for the selection of highly effective siRNA sequences for mammalian and chick RNA interference. Nucleic Acids Res..

[44] Reynolds A (2004). Rational siRNA design for RNA interference. Nat. Biotechnol..

[45] Matveeva OV (2010). Optimization of duplex stability and terminal asymmetry for shRNA design. PLoS ONE.

[46] Technote 2: Ways to Reduce siRNA Off-target Effects. https://www.sitoolsbiotech.com/pdf/WaystoReduceofftargets2-181001.pdf. (2021).

[47] Kaczmarek JC, Kowalski PS, Anderson DG (2017). Advances in the delivery of RNA therapeutics: From concept to clinical reality. Genome Med..

[48] Gane EJ (2017). Future anti-HBV strategies. Liver Int..

[49] Thi EP (2015). Lipid nanoparticle siRNA treatment of Ebola-virus-Makona-infected nonhuman primates. Nature.

[50] Gottlieb J (2016). ALN-RSV01 for prevention of bronchiolitis obliterans syndrome after respiratory syncytial virus infection in lung transplant recipients. J. Heart Lung Transplant..

[51] Hodgson J (2020). The pandemic pipeline. Nat. Biotechnol..

[52] Reischl D, Zimmer A (2009). Drug delivery of siRNA therapeutics: potentials and limits of nanosystems. Nanomedicine.

[53] Zhang S, Zhao B, Jiang H, Wang B, Ma B (2007). Cationic lipids and polymers mediated vectors for delivery of siRNA. J. Control. Rel..

[54] Alagia A, Eritja R (2016). siRNA and RNAi optimization. Wiley Interdiscip. Rev. RNA.

[55] Lei X (2020). Activation and evasion of type I interferon responses by SARS-CoV-2. Nat. Commun..

[56] Parlikar A (2020). Understanding genomic diversity, pan-genome, and evolution of SARS-CoV-2. PeerJ.

[57] Wang C (2020). The establishment of reference sequence for SARS-CoV-2 and variation analysis. J. Med. Virol..

[58] Elbashir SM, Martinez J, Patkaniowska A, Lendeckel W, Tuschl T (2001). Functional anatomy of siRNAs for mediating efficient RNAi in Drosophila melanogaster embryo lysate. EMBO J..

[59] Takasaki S, Kotani S, Konagaya A (2004). An effective method for selecting siRNA target sequences in mammalian cells. Cell Cycle.

[60] Saetrom P, Snøve O (2004). A comparison of siRNA efficacy predictors. Biochem. Biophys. Res. Commun..

[61] Shabalina SA, Spiridonov AN, Ogurtsov AY (2006). Computational models with thermodynamic and composition features improve siRNA design. BMC Bioinform..

[62] de Carli GJ (2020). SSD—a free software for designing multimeric mono-, bi- and trivalent shRNAs. Genet. Mol. Biol..

[63] Biswas SK, Mudi SR (2020). Genetic variation in SARS-CoV-2 may explain variable severity of COVID-19. Med. Hypotheses.

[64] Yeung ML, Bennasser Y, Le SY, Jeang KT (2005). siRNA, miRNA and HIV: Promises and challenges. Cell Res..

[65] Yin Y (2013). Asymmetric siRNA targeting the bcl-2 gene inhibits the proliferation of cancer cells in vitro and in vivo. Int. J. Oncol..

[66] siRNA Design Guidelines | Technical Bulletin #506 - BR. https://www.thermofisher.com/br/en/home/references/ambion-tech-support/rnai-sirna/general-articles/-sirna-design-guidelines.html. (2020).

[67] Langmead B, Trapnell C, Pop M, Salzberg SL (2009). Ultrafast and memory-efficient alignment of short DNA sequences to the human genome. Genome Biol..

[68] Kibbe WA (2007). OligoCalc: An online oligonucleotide properties calculator. Nucleic Acids Res..

