# Supplementary material for: A small interfering RNA (siRNA) database for SARS-CoV-2
Source: Sci Rep. 2021 Apr 23;11:8849. doi: 10.1038/s41598-021-88310-8 (PMC8065152; doi:10.1038/s41598-021-88310-8)
Supplement: Supplementary file 1 — Supplementary Information [file 41598_2021_88310_MOESM1_ESM.docx]

**A small interfering RNA (siRNA) database for SARS-CoV-2**

Inácio Gomes Medeiros^1,3^, André Salim Khayat^5^, Beatriz Stransky^2,4^, Sidney Santos^5^, Paulo Assumpção^6^ and Jorge Estefano Santana de Souza^1,2,^*

^1^ Bioinformatics Graduate Program, Metrópole Digital Institute, Federal University of Rio Grande do Norte, Natal, Rio Grande do Norte, 59078-400, Brazil

^2^ Bioinformatics Multidisciplinary Environment (BioME), Metrópole Digital Institute, Federal University of Rio Grande do Norte, Natal, Rio Grande do Norte, 59078-400, Brazil

^3^ Instituto do Cérebro, Federal University of Rio Grande do Norte, Natal, Rio Grande do Norte, 59078-970, Brazil

^4^ Biomedical Engineering Department, Center of Technology, Federal University of Rio Grande do Norte, Natal, Rio Grande do Norte, 59078-970, Brazil

^5^ Instituto de Ciências Biológicas, Universidade Federal do Pará, Belém, Pará, 66075-110, Brazil

^6^ Núcleo de Pesquisas em Oncologia, Universidade Federal do Pará, Belém, Pará, 66073-110, Brazil

* To whom correspondence should be addressed. Tel: +558497085398; Email: [jorge@imd.ufrn.br](mailto:jorge@imd.ufrn.br)

**Supplementary Data**

Supplementary Text S1 - Features description

A set of more than 170 features was calculated for each sequence from the *target region* set together with their derivations in seven new sets (see Methods section). In this supplementary text, we describe each column of the proposed database, as well as its context and importance.

*Sequence sets*

The first column, *Seq_id*, provides the siRNA identifier, while the second column, *Target_region*, displays the corresponding nucleotide sequence in the SARS-CoV-2 genome that is intended to be targeted by siRNA.

Columns *Nat_Sense*, o*Nat_Sense*, *Sin_Sense*, o*Sin_Sense*, *Antisense*, *oAntisense*, and *oAntisense* (Spreadsheet columns D, E, G, H, J, K, L, see Supplementary Table S1) provide, respectively, corresponding *natural sense*, *oligo natural sense*, *synthetic sense*, *oligo synthetic sense*, *antisense*, *oligo antisense*, and *oligo antisense rev* sequences to *Target_region* sequence. *oligo synthetic sense*, *oligo antisense*, and *oligo antisense rev* are the RNA sequences that can be used to factoring siRNAs. *synthetic sense* and *antisense* (this last for both *oligo antisense* and *oligo antisense rev*) are their respectively DNA counterparts. *natural sense* and *oligo natural sense* are, respectively, the corresponding cDNA and RNA stretches that would be targeted by corresponding produced siRNA.

Column *Annot* indicates which SARS-CoV-2 genes sequences in previous columns come from, and so can be used to select siRNAs that target a specific gene of interest, being 1 if it starts, 0 otherwise (or even more than one, because many SARS-CoV-2 genes overlap among themselves).

*Sequence features*

According to the literature regarding siRNA efficiency[^1–3^](https://paperpile.com/c/1mHqn6/Wo8k+YsnI+Zabj), the base composition of siRNA’s sense and antisense strands has an impact on siRNA efficiency. Some of the criteria reported by cited studies state that effective siRNAs should have (1) A or U at the 5’ end of the antisense strand, and G or C at the 5’ end of the sense strand; (2) GC% content should be low[^4^](https://paperpile.com/c/1mHqn6/G3QE), between 36% and 52%[^5^](https://paperpile.com/c/1mHqn6/Tb7J); (3) an AU-rich 7 bp region at 5’ end of antisense strand[^1^](https://paperpile.com/c/1mHqn6/Wo8k); (4) absence of tetranucleotides UUUU (because it is an RNA-Polymerase tag) and GCCA (considered a toxic tag[^6^](https://paperpile.com/c/1mHqn6/FKyk)), so as AU-rich pentamers, and palindromic regions[^4^](https://paperpile.com/c/1mHqn6/G3QE). However different applications aside from human health context may impose different requirements. Therefore, the following columns are available so that users can make such assessments and select siRNAs with higher efficiency following their own customized filters.

Columns *sAA*, *sTT*, *sGG*, and *sCC* mark whether *Target_region* sequence starts with AA, TT, GG, or CC, respectively, and columns *fAA*, *fTT*, *fCC*, and *fGG*, whether it ends with (1 if so, 0 otherwise).

Columns *A*, *U*, *G*, *C*, *GC*, *AU*, *UUUU*, *GCCA*, and *QtdPenta80%* contains the following information for *oligo natural sense* sequence: quantities of adenine, uracil, guanine, and cytosine; GC% and AU% content; whether tetranucleotides UUUU and GCCA, respectively, are present in sequence (1 for the present, 0 otherwise); the number of pentamers (in the sequence, obtained through a traversing over the sequence with a 5-long one step sliding window) that are AU-rich (80% of it is AU). These same features are also calculated for *oligo synthetic sense* sequences (Spreadsheet columns AL to AT, see Supplementary Table S1) and *oligo antisense* ones (Spreadsheet columns AW to BE). Column *hepta_Sense* AU% content of the first heptamer from *oligo antisense rev* sequence, while column *hepta_AS* gives GC% content from the last heptamer of it.

Columns *Palindromic_N*, *Palindromic_S*, and *Palindromic_AS* indicate if there are any 6-length palindromic regions (1 for true, 0 otherwise) in *natural sense*, *synthetic sense*, and *antisense* sequences, respectively.

*Viruses and SARS-CoV-2 strains coverages*

Given the genetic diversity of SARS-CoV-2, which was enhanced since COVID-19 turned into a pandemic, it is natural that a given siRNA may be poorly effective against a specific set of SARS-CoV-2 strains, while highly effective in another, because of the possible variants that appeared during the spread of the virus. On the other hand, this same siRNA could also have highly effective activity against SARS-CoV and other viruses, due to viruses’ genetic similarity. Another issue regarding genetic similarity is the possible match with the human genome with potential unintended and negative consequences . In order to be possible to make these assessments, the following columns provide coverage information of SARS-CoV-2 siRNAs against strains from diverse countries, as well as genomes of related viruses, human genome, human coding transcriptome, and human non-coding transcriptome.

Columns *hs*, *hs_cds*, *hs_ncrna*, *mers*, *sars*, *h1n1* presents the minimum number of mismatches needed for *natural sense* sequence to match with the human genome (NCBI accession code GRCh37) and coding and non-coding transcriptome, MERS genome (NCBI accession code MG987420), SARS genome (NCBI accession code NC_004718), and Influenza virus genome (NCBI accession code NC_026438). On the other hand, columns *Brazil (57)*, *Wuhan (48)*, *China (41)*, *England (3416)*, *Germany (180)*, *Italy (82)*, *Russia (154)*, *Spain (410)*, and *USA (4725)* display the respective number of SARS-CoV-2 strains genomes (the header of each column displays the total number of strains from each country that sequences were aligned to) from Brazil, China (Wuhan region only), China (without Wuhan region), England, Germany, Italy, Russia, Spain and USA that *natural sense* sequence has a perfect match with. These same features are also calculated for *synthetic sense* sequences (Spreadsheet columns CG to CV) and *antisense* ones (Spreadsheet columns CY to DN).

*Thermodynamic information and efficiency/efficacy prediction*

Thermodynamic information also plays an important role in determining siRNAs efficiency, together with sequence features already discussed above. It is considered that effective siRNA activity occurs when its antisense strand is the one loaded into RISC[^7,8^](https://paperpile.com/c/1mHqn6/av9t+lbzE), being the “selection” of which strand will be loaded is made upon the thermodynamic differences of the two strands’ 5’ end base-pairing stabilities[^9^](https://paperpile.com/c/1mHqn6/cwjW).

It is also reported[^1,10^](https://paperpile.com/c/1mHqn6/Wo8k+WTkki) that efficient siRNAs duplexes display less stable interactions at the 5’ ends of antisense strands and more stable at the 3’ ends. siRNA efficiency-prediction softwares[^10–12^](https://paperpile.com/c/1mHqn6/2Uqj+WTkki+zhesU) exploited this information for efficiency assessment, which emphasizes their importance. However, there is no consensus about siRNA efficiency based on thermodynamic features. Different works[^12–14^](https://paperpile.com/c/1mHqn6/zhesU+UNk8+PbzY) identify different ranges (OF WHAT??) for measuring siRNA general structure stability (with a suggestion about its efficiency), such that the same siRNA may be considered as efficient by one tool and inefficient by another. For example, while Matveeva *et al*[*^12^*](https://paperpile.com/c/1mHqn6/zhesU) reports a range of -32 to -28 kcal/mol like a sign of efficiency, Pereira *et al*[*^14^*](https://paperpile.com/c/1mHqn6/PbzY) works with evaluation values between -9.3 and 9.3 kcal/mol, setting a threshold of efficiency to 6 kcal/mol.

To get around these issues, the following columns provide thermodynamic information and efficiency prediction from diverse tools[^10–12,15^](https://paperpile.com/c/1mHqn6/0GZE+2Uqj+WTkki+zhesU) . Therefore, users that may be in contact with multiple interpretations about efficiency of their siRNAs of interests, and their thermodynamic context, may choose the best ones that meet their specific requirements.

Columns *Tm*, *TmSalt*, *TmNN*, *RlogK*, *deltaG*, *deltaH*, *deltaS*, *Hairpin*, *SelfAnnealing*, and *3’ comp* bring thermodynamic information provided by OligoCalc[^15^](https://paperpile.com/c/1mHqn6/0GZE) to *oligo natural sense*, namely: melting temperature, melting temperature adjusted considering a Na^+^ concentration of 50mM, melting temperature calculated as described in[^16^](https://paperpile.com/c/1mHqn6/vtM9F) using the values available in[^17^](https://paperpile.com/c/1mHqn6/QqP3n), the product between general gas constant *R* and the natural logarithm of 1 over primer concentration, sequence ΔG (change of oligonucleotide’s free energy), sequence ΔH (change of enthalpy), sequence ΔS (change of entropy), number of potential hairpin sites, number of potential self-annealing sites, and whether sequence 3’ have self-complementarity (1 if yes, 0 otherwise). These same features are also calculated for *oligo synthetic sense* sequences (Spreadsheet columns EC to EL) and *oligo antisense* ones (Spreadsheet columns EO to EX). In order to apply it to a huge volume of sequences, we have translated javascript code of OligoCalc web server related to thermodynamic information calculus to in-house Python (https://www.python.org) scripts.

The next columns (Spreadsheet columns FA to FJ) bring efficiency score and thermodynamic information provided by *ThermoComposition21* program[^10^](https://paperpile.com/c/1mHqn6/WTkki) to *natural sense* sequence, namely: *Predicted Eficacy*, as the name suggests, informs the siRNA predicted efficacy, quantified by its gene silenced activity, ranging from 0 (complete gene knockout) to 100 (no effect); *#GG*, the number of GG dinucleotides present in the sequence; *dG(-1)* and *dG(-2..-7)*, “the stability profile (∆G) of each two neighboring base pairs in the siRNA sense-antisense”[^10^](https://paperpile.com/c/1mHqn6/WTkki) at position 1 and at the region from position 2 to position 7, respectively; *dG(2.6.13)*, “the stability (∆G) of dimers of siRNAs antisense strands”[^10^](https://paperpile.com/c/1mHqn6/WTkki); *dG_Best*, “the number of potential target copies in mRNAs (∆G threshold)”[^10^](https://paperpile.com/c/1mHqn6/WTkki); *dG target*, “local target mRNA stabilities (∆G)”[^10^](https://paperpile.com/c/1mHqn6/WTkki); *dG duplex*, “∆G of sense-antisense siRNA duplexes”[^10^](https://paperpile.com/c/1mHqn6/WTkki); *dG(18)*, “∆G difference between position 1 and 18”[^10^](https://paperpile.com/c/1mHqn6/WTkki); and *dG_self*, “antisense strand intra-molecular structure stability (∆G)”[^10^](https://paperpile.com/c/1mHqn6/WTkki). These same features are also calculated by the mentioned tool for *synthetic sense* sequences (Spreadsheet columns FM to FV) and *antisense* ones (Spreadsheet columns FY to GH).

Column *GOOD* (Spreadsheet column GJ) provides predicted efficiency (categorical field, where 1 means that sequence in question is an efficient siRNA, 0 otherwise) from SSD program[^11^](https://paperpile.com/c/1mHqn6/2Uqj) over *natural sense* sequence. The next columns (Spreadsheet columns GL to GO) bring efficiency score and thermodynamic information provided by the tool, namely: *DDG*, the difference (in ∆G) of *DGss* and *Dgem* properties; *DGss*, the structure stability (∆G) of first five nucleotides; *Dgem*, the structure stability (∆G) of first five nucleotides of the sequence’s reverse complement; and *DG*, the sequence structure stability (∆G). These same features are also calculated by the mentioned tool for *synthetic sense* sequences (Spreadsheet columns GR to GU) and *antisense* ones (Spreadsheet columns GX to HA).

Finally, columns *GOOD*, *DG*, and *DDG* (Spreadsheet columns HI to HK) bring efficiency prediction and thermodynamic information provided by software *si-shRNA Selector* program[^12^](https://paperpile.com/c/1mHqn6/zhesU) to *natural sense* sequence, respectively: predicted efficiency (categorical field, where 1 means that sequence in question is an efficient siRNA, 0 otherwise), sequence structure stability (∆G), and terminal duplex asymmetry (∆∆G). These same features are also calculated by the mentioned tool for *synthetic sense* sequences (Spreadsheet columns HI to HK) and *antisense* ones (Spreadsheet columns HN to HP).

References

1. [Ui-Tei, K. *et al.* Guidelines for the selection of highly effective siRNA sequences for mammalian and chick RNA interference. *Nucleic Acids Res.* **32**, 936–948 (2004).](http://paperpile.com/b/1mHqn6/Wo8k)

2. [Huesken, D. *et al.* Design of a genome-wide siRNA library using an artificial neural network. *Nat. Biotechnol.* **23**, 995–1001 (2005).](http://paperpile.com/b/1mHqn6/YsnI)

3. [siRNA Design Guidelines | Technical Bulletin #506 - BR. *https://www.thermofisher.com/br/en/home/references/ambion-tech-support/rnai-sirna/general-articles/-sirna-design-guidelines.html* (2020).](http://paperpile.com/b/1mHqn6/Zabj)

4. [Technote 2: Ways to Reduce siRNA Off-target Effects. *https://www.sitoolsbiotech.com/pdf/WaystoReduceofftargets2-181001.pdf* (2021).](http://paperpile.com/b/1mHqn6/G3QE)

5. [Reynolds, A., Leake, D., Boese, Q. & Scaringe, S. Rational siRNA design for RNA interference. *Nature* (2004).](http://paperpile.com/b/1mHqn6/Tb7J)

6. [Fedorov, Y. *et al.* Off-target effects by siRNA can induce toxic phenotype. *RNA* **12**, 1188–1196 (2006).](http://paperpile.com/b/1mHqn6/FKyk)

7. [Khvorova, A., Reynolds, A. & Jayasena, S. D. Functional siRNAs and miRNAs exhibit strand bias. *Cell* **115**, 209–216 (2003).](http://paperpile.com/b/1mHqn6/av9t)

8. [Schwarz, D. S. *et al.* Asymmetry in the assembly of the RNAi enzyme complex. *Cell* **115**, 199–208 (2003).](http://paperpile.com/b/1mHqn6/lbzE)

9. [Tomari, Y., Matranga, C., Haley, B., Martinez, N. & Zamore, P. D. A protein sensor for siRNA asymmetry. *Science* **306**, 1377–1380 (2004).](http://paperpile.com/b/1mHqn6/cwjW)

10. [Shabalina, S. A., Spiridonov, A. N. & Ogurtsov, A. Y. Computational models with thermodynamic and composition features improve siRNA design. *BMC Bioinformatics* **7**, 65 (2006).](http://paperpile.com/b/1mHqn6/WTkki)

11. [Carli, G. J. de *et al.* SSD - a free software for designing multimeric mono-, bi- and trivalent shRNAs. *Genet. Mol. Biol.* **43**, e20190300 (2020).](http://paperpile.com/b/1mHqn6/2Uqj)

12. [Matveeva, O. V. *et al.* Optimization of duplex stability and terminal asymmetry for shRNA design. *PLoS One* **5**, e10180 (2010).](http://paperpile.com/b/1mHqn6/zhesU)

13. [Ichihara, M. *et al.* Thermodynamic instability of siRNA duplex is a prerequisite for dependable prediction of siRNA activities. *Nucleic Acids Res.* **35**, e123 (2007).](http://paperpile.com/b/1mHqn6/UNk8)

14. [Pereira, T. C. *et al.* Strand Analysis, a free online program for the computational identification of the best RNA interference (RNAi) targets based on Gibbs free energy. *Genet. Mol. Biol.* **30**, 1206–1208 (2007).](http://paperpile.com/b/1mHqn6/PbzY)

15. [Kibbe, W. A. OligoCalc: an online oligonucleotide properties calculator. *Nucleic Acids Res.* **35**, W43–6 (2007).](http://paperpile.com/b/1mHqn6/0GZE)

16. [Breslauer, K. J., Frank, R., Blöcker, H. & Marky, L. A. Predicting DNA duplex stability from the base sequence. *Proc. Natl. Acad. Sci. U. S. A.* **83**, 3746–3750 (1986).](http://paperpile.com/b/1mHqn6/vtM9F)

17. [Sugimoto, N., Nakano, S., Yoneyama, M. & Honda, K. Improved thermodynamic parameters and helix initiation factor to predict stability of DNA duplexes. *Nucleic Acids Res.* **24**, 4501–4505 (1996).](http://paperpile.com/b/1mHqn6/QqP3n)

**Supplementary Table S1 - Database columns descriptions.** The description of each column of proposed database. This structure is the same for the four spreadsheet and CSV files. Text in asterisk was taken (and slightly adapted) from (4)

| **Spreadsheet Column** | **Column** | **Description** | **Example value** |
| --- | --- | --- | --- |
| A | Seq_id | Sequence identifier | Seq_100 |
| B | Target_region | Sequence of SARS-CoV-2’s target region | 5'-GGCTGCATGCTTAGTGCACTCAC-3' |
| D | Nat_Sense | siRNA’s natural sense sequence from of SARS-CoV-2’s target region | 5'-CTGCATGCTTAGTGCACTCAC-3' |
| E | oNat_Sense | siRNA’s natural sense oligonucleotide sequence from of SARS-CoV-2’s target region | 5'-CUGCAUGCUUAGUGCACUCAC-3' |
| G | Sin_Sense | siRNA’s synthetic sense sequence from of SARS-CoV-2’s target region | 5'-CTGCATGCTTAGTGCACTCTT-3' |
| H | oSin_Sense | siRNA’s synthetic sense oligonucleotide sequence from of SARS-CoV-2’s target region | 5'-CUGCAUGCUUAGUGCACUCUU-3' |
| J | AntiSense | siRNA’s antisense sequence from of SARS-CoV-2’s target region | 3'-CCGACGTACGAATCACGTGAG-5' |
| K | oAntiSense | siRNA’s antisense oligonucleotide sequence from of SARS-CoV-2’s target region | 3'-CCGACGUACGAAUCACGUGAG-5' |
| L | oAntiSense | siRNA’s oAntiSense (reverse) oligonucleotide sequence from of SARS-CoV-2’s target region | 5'-GAGUGCACUAAGCAUGCAGCC-3' |
| N | Annot | SARS-CoV-2 genes contemplated in Target_region sequence | -- |
| P | sAA | It indicates whether the sequence from Target_region starts with AA (1 if it  starts, 0 otherwise) | 0 |
| Q | sTT | It indicates whether the sequence from Target_region starts with TT (1 if it  starts, 0 otherwise) | 0 |
| R | sGG | It indicates whether the sequence from Target_region starts with GG (1 if it  starts, 0 otherwise) | 1 |
| S | sCC | It indicates whether the sequence from Target_region starts with CC (1 if it  starts, 0 otherwise) | 0 |
| U | fTT | It indicates whether the sequence from Target_region ends with TT (1 if it  starts, 0 otherwise) | 0 |
| V | fAA | It indicates whether the sequence from Target_region ends with AA (1 if it  starts, 0 otherwise) | 0 |
| W | fCC | It indicates whether the sequence from Target_region ends with CC (1 if it  starts, 0 otherwise) | 0 |
| X | fGG | It indicates whether the sequence from Target_region ends with GG (1 if it  starts, 0 otherwise) | 0 |
| AA | A | Number of adenines in oNat_Sense sequence | 4 |
| AB | U | Number of uracils in oNat_Sense sequence | 6 |
| AC | G | Number of guanines in oNat_Sense sequence | 4 |
| AD | C | Number of cytosines in oNat_Sense sequence | 7 |
| AE | GC | GC% content of oNat_Sense sequence | 0.52 |
| AF | AU | AU% content of oNat_Sense sequence | 0.48 |
| AG | UUUU | It indicates whether the tetranucleotide UUUU is present in oNat_Sense sequence (1 for present, 0 otherwise) | 0 |
| AH | GCCA | It indicates whether the tetranucleotide GCCA is present in oNat_Sense sequence (1 for present, 0 otherwise) | 0 |
| AI | QtdPenta80% | Number of AU-rich pentamers in oNat_Sense sequence | 1 |
| AL | A | Number of adenines in oSin_Sense sequence | 3 |
| AM | U | Number of uracils in oSin_Sense sequence | 8 |
| AN | G | Number of guanines in oSin_Sense sequence | 4 |
| AO | C | Number of cytosines in oSin_Sense sequence | 6 |
| AP | GC | GC% content of oSin_Sense sequence | 0.48 |
| AQ | AU | AU% content of oSin_Sense sequence | 0.52 |
| AR | UUUU | It indicates whether the tetranucleotide UUUU is present in oSin_Sense sequence (1 for present, 0 otherwise) | 0 |
| AS | GCCA | It indicates whether the tetranucleotide GCCA is present in oSin_Sense sequence (1 for present, 0 otherwise) | 0 |
| AT | QtdPenta80% | Number of AU-rich pentamers in oSin_Sense sequence | 1 |
| AW | A | Number of adenines in oAntiSense sequence | 6 |
| AX | U | Number of uracils in oAntiSense sequence | 3 |
| AY | G | Number of guanines in oAntiSense sequence | 6 |
| AZ | C | Number of cytosines in oAntiSense sequence | 6 |
| BA | GC | GC% content of oAntiSense sequence | 0.57 |
| BB | AU | AU% content of oAntiSense sequence | 0.43 |
| BC | UUUU | It indicates whether the tetranucleotide UUUU is present in oAntiSense sequence (1 for present, 0 otherwise) | 0 |
| BD | GCCA | It indicates whether the tetranucleotide GCCA is present in oAntiSense sequence (1 for present, 0 otherwise) | 0 |
| BE | QtdPenta80% | Number of AU-rich pentamers in oAntiSense sequence | 1 |
| BG | hepta_Sense | AU% content of the first heptamer from oAntiSense (reverse) oligonucleotide sequence | 0.43 |
| BH | hepta_AS | GC% content from the last heptamer from oAntiSense (reverse) oligonucleotide sequence | 0.57 |
| BJ | Palindromic_N | Presence of some 6-length palindromic window (1 for true, 0 Otherwise) in Nat_Sense sequence | 1 |
| BK | Palindromic_S | Presence of some 6-length palindromic window (1 for true, 0 Otherwise) in Sin_Sense sequence | 1 |
| BL | Palindromic_AS | Presence of some 6-length palindromic window (1 for true, 0 Otherwise) in AntiSense sequence | 1 |
| BO | hs | Minimum number of mismatches needed for Nat_Sense sequence have homology with the human genome (NCBI accession code GRCh37) | 2 |
| BP | hs_cds | Minimum number of mismatches needed for Nat_Sense sequence have homology with the human coding transcriptome | 3 |
| BQ | hs_ncrna | Minimum number of mismatches needed for Nat_Sense sequence have homology with the human non-coding transcriptome | 2 |
| BR | mers | Minimum number of mismatches needed for Nat_Sense sequence have homology with MERS genome (NCBI accession code MG987420) | 6 |
| BS | sars | Minimum number of mismatches needed for Nat_Sense sequence have homology with SARS genome (NCBI accession code NC_004718) | 3 |
| BT | h1n1 | Minimum number of mismatches needed for Nat_Sense sequence have homology with SARS genome (NCBI accession code NC_026438) | 5 |
| BV | Brazil (57) | Minimum number of mismatches needed for Nat_Sense sequence have homology with 57 SARS-CoV-2 strains from Brazil | 49 |
| BW | Wuhan (39) | Minimum number of mismatches needed for Nat_Sense sequence have homology with 39 SARS-CoV-2 strains from Wuhan | 35 |
| BX | China (23) | Minimum number of mismatches needed for Nat_Sense sequence have homology with 23 SARS-CoV-2 strains from China | 23 |
| BY | England (3012) | Minimum number of mismatches needed for Nat_Sense sequence have homology with 3012 SARS-CoV-2 strains from England | 3385 |
| BZ | Germany (180) | Minimum number of mismatches needed for Nat_Sense sequence have homology with 180 SARS-CoV-2 strains from Germany | 180 |
| CA | Italy (77) | Minimum number of mismatches needed for Nat_Sense sequence have homology with 77 SARS-CoV-2 strains from Italy | 75 |
| CB | Russia (157) | Minimum number of mismatches needed for Nat_Sense sequence have homology with 157 SARS-CoV-2 strains from Russia | 139 |
| CC | Spain (410) | Minimum number of mismatches needed for Nat_Sense sequence have homology with 410 SARS-CoV-2 strains from Spain | 410 |
| CD | USA (4724) | Minimum number of mismatches needed for Nat_Sense sequence have homology with 4724 SARS-CoV-2 strains from USA | 4357 |
| CG | hs | Minimum number of mismatches needed for Sin_Sense sequence have homology with the human genome (NCBI accession code GRCh37) | 1 |
| CH | hs_cds | Minimum number of mismatches needed for Sin_Sense sequence have homology with the human coding transcriptome | 4 |
| CI | hs_ncrna | Minimum number of mismatches needed for Sin_Sense sequence have homology with the human non-coding transcriptome | 4 |
| CJ | mers | Minimum number of mismatches needed for Sin_Sense sequence have homology with MERS genome (NCBI accession code MG987420) | 6 |
| CK | sars | Minimum number of mismatches needed for Sin_Sense sequence have homology with SARS genome (NCBI accession code NC_004718) | 5 |
| CL | h1n1 | Minimum number of mismatches needed for Sin_Sense sequence have homology with SARS genome (NCBI accession code NC_026438) | 4 |
| CN | Brazil (57) | Minimum number of mismatches needed for Nat_Sense sequence have homology with 57 SARS-CoV-2 strains from Brazil | 0 |
| CO | Wuhan (39) | Minimum number of mismatches needed for Nat_Sense sequence have homology with 39 SARS-CoV-2 strains from Wuhan | 0 |
| CP | China (23) | Minimum number of mismatches needed for Nat_Sense sequence have homology with 23 SARS-CoV-2 strains from China | 0 |
| CQ | England (3012) | Minimum number of mismatches needed for Nat_Sense sequence have homology with 3012 SARS-CoV-2 strains from England | 0 |
| CR | Germany (180) | Minimum number of mismatches needed for Nat_Sense sequence have homology with 180 SARS-CoV-2 strains from Germany | 0 |
| CS | Italy (77) | Minimum number of mismatches needed for Nat_Sense sequence have homology with 77 SARS-CoV-2 strains from Italy | 0 |
| CT | Russia (157) | Minimum number of mismatches needed for Nat_Sense sequence have homology with 157 SARS-CoV-2 strains from Russia | 0 |
| CU | Spain (410) | Minimum number of mismatches needed for Nat_Sense sequence have homology with 410 SARS-CoV-2 strains from Spain | 0 |
| CV | USA (4724) | Minimum number of mismatches needed for Nat_Sense sequence have homology with 4724 SARS-CoV-2 strains from USA | 0 |
| CY | hs | Minimum number of mismatches needed for AntiSense sequence have homology with the human genome (NCBI accession code GRCh37) | 2 |
| CZ | hs_cds | Minimum number of mismatches needed for AntiSense sequence have homology with the human coding transcriptome | 4 |
| DA | hs_ncrna | Minimum number of mismatches needed for AntiSense sequence have homology with the human non-coding transcriptome | 3 |
| DB | mers | Minimum number of mismatches needed for AntiSense sequence have homology with MERS genome (NCBI accession code MG987420) | 5 |
| DC | sars | Minimum number of mismatches needed for AntiSense sequence have homology with SARS genome (NCBI accession code NC_004718) | 3 |
| DD | h1n1 | Minimum number of mismatches needed for AntiSense sequence have homology with SARS genome (NCBI accession code NC_026438) | 6 |
| DF | Brazil (57) | Minimum number of mismatches needed for Nat_Sense sequence have homology with 57 SARS-CoV-2 strains from Brazil | 49 |
| DG | Wuhan (39) | Minimum number of mismatches needed for Nat_Sense sequence have homology with 39 SARS-CoV-2 strains from Wuhan | 35 |
| DH | China (23) | Minimum number of mismatches needed for Nat_Sense sequence have homology with 23 SARS-CoV-2 strains from China | 23 |
| DI | England (3012) | Minimum number of mismatches needed for Nat_Sense sequence have homology with 3012 SARS-CoV-2 strains from England | 3385 |
| DJ | Germany (180) | Minimum number of mismatches needed for Nat_Sense sequence have homology with 180 SARS-CoV-2 strains from Germany | 180 |
| DK | Italy (77) | Minimum number of mismatches needed for Nat_Sense sequence have homology with 77 SARS-CoV-2 strains from Italy | 75 |
| DL | Russia (157) | Minimum number of mismatches needed for Nat_Sense sequence have homology with 157 SARS-CoV-2 strains from Russia | 139 |
| DM | Spain (410) | Minimum number of mismatches needed for Nat_Sense sequence have homology with 410 SARS-CoV-2 strains from Spain | 409 |
| DN | USA (4724) | Minimum number of mismatches needed for Nat_Sense sequence have homology with 4724 SARS-CoV-2 strains from USA | 4356 |
| **Features calculated with OligoCalc (1)** | | | |
| DQ | Tm | Melting temperature of oNat_Sense sequence | 54 |
| DR | TmSalt | Melting temperature of oNat_Sense sequence adjusted considering a Na + concentration of 50mM | 50 |
| DS | TmNN | Melting temperature of oNat_Sense sequence calculated as described in (2) using the values available in (3) | 65 |
| DT | RlogK | Product between general gas  constant R and natural logarithm of 1 over primer concentration of oNat_Sense sequence | 33404 |
| DU | deltaG | oNat_Sense sequence ΔG (change of oligonucleotide’s free energy) | 27.1 |
| DV | deltaH | oNat_Sense sequence ΔH (change of enthalpy) | 219.71 |
| DW | deltaS | oNat_Sense sequence ΔS (change of entropy) | 567.7 |
| DX | Hairpin | Number of potential hairpin sites of oNat_Sense sequence | 0 |
| DY | SelfAnnealing | Number of potential self-annealing sites in oNat_Sense sequence | 0 |
| DZ | 3´comp | It indicates whether oNat_Sense sequence 3’ have self-complementarity (1 if yes, 0 otherwise) | 0 |
| EC | Tm | Melting temperature of oSin_Sense sequence | 52 |
| ED | TmSalt | Melting temperature of oSin_Sense sequence adjusted considering a Na+ concentration of 50mM | 47 |
| EE | TmNN | Melting temperature of oSin_Sense sequence calculated as described in (2) using the values available in (3) | 64 |
| EF | RlogK | Product between general gas  constant R and natural logarithm of 1 over primer concentration of oSin_Sense sequence | 33404 |
| EG | deltaG | oSin_Sense sequence ΔG (change of oligonucleotide’s free energy) | 26.6 |
| EH | deltaH | oSin_Sense sequence ΔH (change of enthalpy) | 215.15 |
| EI | deltaS | oSin_Sense sequence ΔS (change of entropy) | 557.4 |
| EJ | Hairpin | Number of potential hairpin sites of oSin_Sense sequence | 0 |
| EK | SelfAnnealing | Number of potential self-annealing sites in oSin_Sense sequence | 0 |
| EL | 3´comp | It indicates whether oSin_Sense sequence 3’ have self-complementarity (1 if yes, 0 otherwise) | 0 |
| EO | Tm | Melting temperature of oAntiSense sequence | 56 |
| EP | TmSalt | Melting temperature of oAntiSense sequence adjusted considering a Na+ concentration of 50mM | 54 |
| EQ | TmNN | Melting temperature of oAntiSense sequence calculated as described in (2) using the values available in (3) | 68 |
| ER | RlogK | Product between general gas  constant R and natural logarithm of 1 over primer concentration of oAntiSense sequence | 33404 |
| ES | deltaG | oAntiSense sequence ΔG (change of oligonucleotide’s free energy) | 28.3 |
| ET | deltaH | oAntiSense sequence ΔH (change of enthalpy) | 226.14 |
| EU | deltaS | oAntiSense sequence ΔS (change of entropy) | 580.9 |
| EV | Hairpin | Number of potential hairpin sites of oAntiSense sequence | 0 |
| EW | SelfAnnealing | Number of potential self-annealing sites in oAntiSense sequence | 0 |
| EX | 3´comp | It indicates whether oAntiSense sequence 3’ have self-complementarity (1 if yes, 0 otherwise) | 0 |
| **Features calculated with ThermoCompisiton21 (4)** | | | |
| **Excel Column** | **Column** | **Description** | **Example value** |
| FA | Predicted Eficacy | Predicted eficacy of Nat_Sense sequence, quantified by its gene silenced activity, ranging from 0 (complete gene knockout) to 100 (no effect) | 6.64 |
| FB | #GG | Number of GG dinucleotides present in Nat_Sense sequence | 0 |
| FC | dG(-1) | Stability profile (∆G) of each two neighboring base pairs in the siRNA sense-antisense at position 1 of Nat_Sense sequence* | 0 |
| FD | dG(-2..-7) | Stability profile (∆G) or each two neighboring base pairs in the siRNA sense-antisense from position 2 to position 7 of Nat_Sense sequence* | 0 |
| FE | dG(2.6.13) | Stability (∆G) of dimers of siRNAs antisense strands of Nat_Sense sequence* | -6.3 |
| FF | dG_Best | Number of potential target copies in mRNAs (∆G threshold) of Nat_Sense sequence* | 0.0 |
| FG | dG target | Local target mRNA stabilities (∆G) for Nat_Sense sequence* | -11.4 |
| FH | dG duplex | ∆G of sense-antisense siRNA duplexes for Nat_Sense sequence | -39.6 |
| FI | dG(18) | ∆G difference between position 1 and 18 in Nat_Sense sequence | -2.4 |
| FJ | dG_self | AntiSense strand intra-molecular structure stability (∆G) of Nat_Sense sequence* | -4.3 |
| FM | Predicted Eficacy | Predicted eficacy of Sin_Sense sequence, quantified by its gene silenced activity, ranging from 0 (complete gene knockout) to 100 (no effect) | 6.66 |
| FN | #GG | Number of GG dinucleotides present in Sin_Sense sequence | 0 |
| FO | dG(-1) | Stability profile (∆G) or each two neighboring base pairs in the siRNA sense-antisense at position 1 of Sin_Sense sequence* | 0 |
| FP | dG(-2..-7) | Stability profile (∆G) or each two neighboring base pairs in the siRNA sense-antisense from position 2 to position 7 of Sin_Sense sequence* | 0 |
| FQ | dG(2.6.13) | Stability (∆G) of dimers of siRNAs antisense strands of Sin_Sense sequence* | -6.3 |
| FR | dG_Best | Number of potential target copies in mRNAs (∆G threshold) of Sin_Sense sequence* | -7.6 |
| FS | dG target | Local target mRNA stabilities (∆G) for Sin_Sense sequence* | -11.2 |
| FT | dG duplex | ∆G of sense-antisense siRNA duplexes for Sin_Sense sequence | -39.6 |
| FU | dG(18) | ∆G difference between position 1 and 18 in Sin_Sense sequence | -2.4 |
| FV | dG_self | AntiSense strand intra-molecular structure stability (∆G) of Sin_Sense sequence* | -4.3 |
| FY | Predicted Eficacy | Predicted eficacy of AntiSense sequence, quantified by its gene silenced activity, ranging from 0 (complete gene knockout) to 100 (no effect) | 6.54 |
| FZ | #GG | Number of GG dinucleotides present in AntiSense sequence | 0 |
| GA | dG(-1) | Stability profile (∆G) or each two neighboring base pairs in the siRNA sense-antisense at position 1 of AntiSense sequence* | 0 |
| GB | dG(-2..-7) | Stability profile (∆G) or each two neighboring base pairs in the siRNA sense-antisense from position 2 to position 7 of AntiSense sequence* | 0 |
| GC | dG(2.6.13) | Stability (∆G) of dimers of siRNAs antisense strands of AntiSense sequence* | -6.3 |
| GD | dG_Best | Number of potential target copies in mRNAs (∆G threshold) of AntiSense sequence* | -7.7 |
| GE | dG target | Local target mRNA stabilities (∆G) for AntiSense sequence* | -11.9 |
| GF | dG duplex | ∆G of sense-antisense siRNA duplexes for AntiSense sequence | -40.6 |
| GG | dG(18) | ∆G difference between position 1 and 18 in AntiSense sequence | -2.1 |
| GH | dG_self | AntiSense strand intra-molecular structure stability (∆G) of AntiSense sequence* | -3.3 |
| **Features calculated with SSD (5)** | | | |
| **Excel Column** | **Column** | **Description** | **Example value** |
| GJ | GOOD | Predicted efficiency, where 1 means that Nat_Sense forms an efficient siRNA, 0 otherwise. | 1 |
| GL | DDG | Difference (in ∆G) of DGss and Dgem variables of Nat_Sense sequence | 0.9 |
| GM | DGss | Structure stability (∆G) of first five nucleotides of Nat_Sense sequence | 10.1 |
| GN | Dgem | Structure stability (∆G) of first five nucleotides of reverse complement of Nat_Sense sequence | 9.2 |
| GO | DG | Nat_Sense sequence structure stability (∆G) | 45.6 |
| GR | DDG | Difference (in ∆G) of DGss and Dgem variables of Sin_Sense sequence | 0.9 |
| GS | DGss | Structure stability (∆G) of first five nucleotides of Sin_Sense sequence | 10.1 |
| GT | Dgem | Structure stability (∆G) of first five nucleotides of reverse complement of Sin_Sense sequence | 9.2 |
| GU | DG | Sin_Sense sequence structure stability (∆G) | 44.0 |
| GX | DDG | Difference (in ∆G) of DGss and Dgem variables of AntiSense sequence | -0.9 |
| GY | DGss | Structure stability (∆G) of first five nucleotides of AntiSense sequence | 9.2 |
| GZ | Dgem | Structure stability (∆G) of first five nucleotides of reverse complement of AntiSense sequence | 10.1 |
| HA | DG | AntiSense sequence structure stability (∆G) | 48.2 |
| **Features calculated with si_shRNA_selector (6)** | | | |
| **Excel Column** | **Column** | **Description** | **Example value** |
| HD | GOOD | Predicted efficiency, where 1 means that Nat_Sense forms an efficient siRNA, 0 otherwise. | 0 |
| HE | DG | Nat_Sense sequence structure stability (∆G) | -37.99 |
| HF | DDG | Terminal duplex asymmetry (∆∆G) of Nat_Sense | 1.43 |
| HI | GOOD | Predicted efficiency, where 1 means that Sin_Sense forms an efficient siRNA, 0 otherwise. | 0 |
| HJ | DG | Sin_Sense sequence structure stability (∆G) | -37.79 |
| HK | DDG | Terminal duplex asymmetry (∆∆G) of Sin_Sense | 1.6 |
| HN | GOOD | Predicted efficiency, where 1 means that AntiSense forms an efficient siRNA, 0 otherwise. | 0 |
| HO | DG | AntiSense sequence structure stability (∆G) | -37.95 |
| HP | DDG | Terminal duplex asymmetry (∆∆G) of AntiSense | 1.87 |

**Supplementary Table S2** **–** **Distribution of siRNAs with high coverage across the genes of SARS-CoV-2**. The numbers of siRNAs that match more than 95% of countries' strains across SARS-CoV-2 genes.

| **Gene** | **Brazil** | **Wuhan** | **China** | **England** | **Germany** | **Italy** | **Russia** | **Spain** | **USA** |
| --- | --- | --- | --- | --- | --- | --- | --- | --- | --- |
| M_protein | 525 | 675 | 655 | 661 | 658 | 656 | 616 | 596 | 679 |
| NC | 194 | 664 | 702 | 711 | 716 | 705 | 712 | 674 | 716 |
| ORF3a | 626 | 789 | 740 | 773 | 794 | 445 | 815 | 510 | 815 |
| S_glycoprotein,Spike_protein_S1 | 1236 | 2019 | 1585 | 1447 | 1473 | 1998 | 1998 | 1506 | 1998 |
| S_glycoprotein,Spike_protein_S2 | 277 | 366 | 266 | 345 | 366 | 366 | 345 | 366 | 366 |
| S_glycoprotein,Spike_protein_S2,Spike_protein_S2' | 1210 | 1364 | 1100 | 1363 | 1385 | 1364 | 1385 | 1385 | 1385 |
| nsp1,pp1ab,pp1a | 519 | 289 | 328 | 540 | 540 | 513 | 527 | 540 | 501 |
| pp1ab,ExoN | 1342 | 1557 | 1441 | 984 | 1242 | 1536 | 1557 | 1557 | 1201 |
| pp1ab,Hel | 1773 | 1779 | 1332 | 1739 | 1779 | 1773 | 1758 | 1779 | 1737 |
| pp1ab,Pol | 2690 | 2732 | 2057 | 2690 | 2690 | 2711 | 2711 | 2565 | 2711 |
| pp1ab,nsp15 | 1014 | 1014 | 650 | 346 | 993 | 1014 | 993 | 993 | 1014 |
| pp1ab,nsp16 | 777 | 894 | 651 | 769 | 862 | 894 | 894 | 894 | 649 |
| pp1ab,pp1a,3CL-PRO | 894 | 894 | 828 | 894 | 894 | 894 | 873 | 894 | 894 |
| pp1ab,pp1a,nsp10 | 393 | 393 | 358 | 393 | 393 | 393 | 393 | 334 | 393 |
| pp1ab,pp1a,nsp2 | 1342 | 1890 | 1241 | 1460 | 1848 | 1869 | 1848 | 1890 | 1869 |
| pp1ab,pp1a,nsp3 | 5039 | 5755 | 3999 | 5480 | 5451 | 5639 | 5769 | 5480 | 5790 |
| pp1ab,pp1a,nsp4 | 1371 | 1455 | 1084 | 1476 | 1476 | 1400 | 1476 | 1196 | 1455 |
| pp1ab,pp1a,nsp6 | 825 | 846 | 685 | 825 | 825 | 825 | 825 | 825 | 833 |
| pp1ab,pp1a,nsp8 | 570 | 570 | 410 | 570 | 570 | 570 | 570 | 570 | 570 |

**Supplementary Table S3.** **List of works about siRNAs applications to SARS-CoV and SARS-CoV-2.** The first column indicates the “label” of each work group. Sixth column shows the number of siRNAs proposed in each work, and the last column, the percentage of them that appear in the proposed database.

|  | **DOI/Link** | **Year** | **Journal** | **Title** | **Number of siRNAs** | **% of siRNAs present in proposed database (D)** |
| --- | --- | --- | --- | --- | --- | --- |
| siRNA computational identification and design papers | 10.1007/s12250-020-00221-6 | 2020 | Virologica Sinica | Computational Identification of Small Interfering RNA Targets in SARS-CoV-2 | 9 | 100% |
|  | 10.1101/2020.04.19.048991 | 2020 | bioRxiv | CoV2ID: Detection and Therapeutics Oligo Database for SARS-CoV-2 | 13 | 100% |
|  | 10.1016/j.ygeno.2020.12.021 | 2021 | Genomics | A computational approach to design potential siRNA molecules as a prospective tool for silencing nucleocapsid phosphoprotein and surface glycoprotein gene of SARS-CoV-2 | 8 | 100% |
|  | 10.1101/2020.07.07.190967 | 2020 | bioRxiv | A Small interfering RNA lead targeting RNA-dependent RNA-polymerase effectively inhibit the SARS-CoV-2 infection in Golden Syrian hamster and Rhesus macaque | 6 | 100% |
|  | 10.1101/2020.08.13.250076 | 2020 | bioRxiv | In Silico design of siRNAs targeting existing and future respiratory viruses with VirusSi (B) | 10 | 100% |
|  | 10.4103/ijmr.IJMR_2855_20 | 2020 | Indian J Med Res | Prediction of potential small interfering RNA molecules for silencing of the spike gene of SARS-CoV-2 (A) | NF | NF |
|  | 10.4149/BLL_2021_035 | 2020 | Bratisl Lek Listy | Potential therapeutic road for targeting the SARS-CoV-2 at throat (A) | NF | NF |
|  | 10.2174/1381612827999210111194101 | 2021 | Curr Pharm Des | In silico Prediction and Designing of Potential siRNAs to be Used as Antivirals Against SARS-CoV-2 (A) | NF | NF |
|  | 10.1002/acg2.107 | 2021 | Advances in Cell and Gene Therapy | An in silico analysis of effective siRNAs against COVID 19 by targeting the leader sequence of SARS CoV 2 | 4 | 100% |
|  | 10.36295/ASRO.2020.231101 | 2020 | Annals of Tropical Medicine and Public Health | An in silico approach to design potential siRNA molecules of SARS-CoV-2 Virus structural genes, a preliminary opinion for COVID-19 inhibition | 52 | 100% |
| Pre-clinical or human-clinical test studies | 10.22541/au.161359798.81563481/v1 | 2021 | Authorea | Silencing of SARS-CoV-2 with modified siRNA-peptide dendrimer formulation | NF | NF |
|  | 10.1101/2020.07.07.190967 | 2020 | bioRxiv | A Small interfering RNA lead targeting RNA-dependent RNA-polymerase effectively inhibit the SARS-CoV-2 infection in Golden Syrian hamster and Rhesus macaque | 6 | 100% |
|  | 10.21203/rs.3.rs-105129/v2 | 2020 | Research Square | Systematic analysis of RNAi-accessible SARS-CoV-2 replication steps identifies ORF1 as promising target | NF | NF |
| Patents submissions | https://patents.google.com/patent/RU2733361C1/en | 2020 | NA | Agent for inhibition of replication of sars-cov-2 virus mediated by rna interference | 30 | 100% |
|  | https://patents.google.com/patent/CN111330003A/en | 2020 | NA | Preparation method of novel coronary pneumonia antisense RNA multivalent vaccine | 43 | 100% |
|  | https://patents.google.com/patent/CN112111489A/en | 2020 | NA | shRNA inhibiting SARS-COV-2 virus replication and its application | 14 | 100% |
|  | https://patents.google.com/patent/CN111321142A/en | 2020 | NA | Preparation method of novel coronavirus pneumonia dsRNA vaccine | NF | NF |
|  | https://patents.google.com/patent/CN111139241A/en | 2020 | NA | Small interfering nucleic acid for inhibiting novel coronavirus, composition and application | 12 | 100% |
|  | https://patents.google.com/patent/CN111139242A/en | 2020 | NA | Small interfering nucleic acid, composition and application | 13 | 100% |
|  | https://patents.google.com/patent/CN111518809A/en | 2020 | NA | siRNA interfering expression of novel coronavirus COVID-19 gene and application thereof | 6 | 100% |
| Related projects | 10.1016/j.gene.2020.145368 | 2021 | Gene | Recognition of plausible therapeutic agents to combat COVID-19: An omics data based combined approach | NA | NA |
|  | 10.14203/beritabiologi.v19i1.3849 | 2020 | BERITA BIOLOGI | The predicted structure for the anti-sense siRNA of the RNA polymerase enzyme (RdRp) gene of the SARS-COV-2 | NA | NA |
|  | https://www.superiorideas.org/projects/silencing-coronavirus | 2020 | NA | Targeting COVID-19 Coronavirus RNA for Degradation by a Nasal Spray of Superior siRNAs | NA | NA |
|  | https://www.alnylam.com/wp-content/uploads/2020/09/OTS-2020_Akinc.pdf | 2020 | NA | ALN-COV: An Investigational RNAi Therapeutic for COVID-19 | NA | NA |
| (A) Authors did not have access to manuscript of this paper | | | | | | |
| (B) Only the reverse-complement of these siRNAs match with the ones from DB | | | | | | |
| (C) Work cited in [10.22541/au.161359798.81563481/v1](http://doi.org/10.22541/au.161359798.81563481/v1) | | | | | | |
| (D) siRNAs from each study were compared with 21nt ones from proposed database. Those with length longer than 21nt were split using a 21-length sliding window, such that comparison could have been performed. It was considered that proposed database have a siRNA if the whole siRNA (or any of the 21nt splits), in either sense or antisense, matches with any siRNA from proposed database. | | | | | | |
| NA - Not applicable | | | | | | |
| NF - Data not found | | | | | | |

**Supplementary Figure S1. Coverage of 21nt siRNAs considered atoxic by literature across strains from nine countries.**

a) Targeting coverage of 21nt siRNAs considered atoxic according to the literature[^1,2^
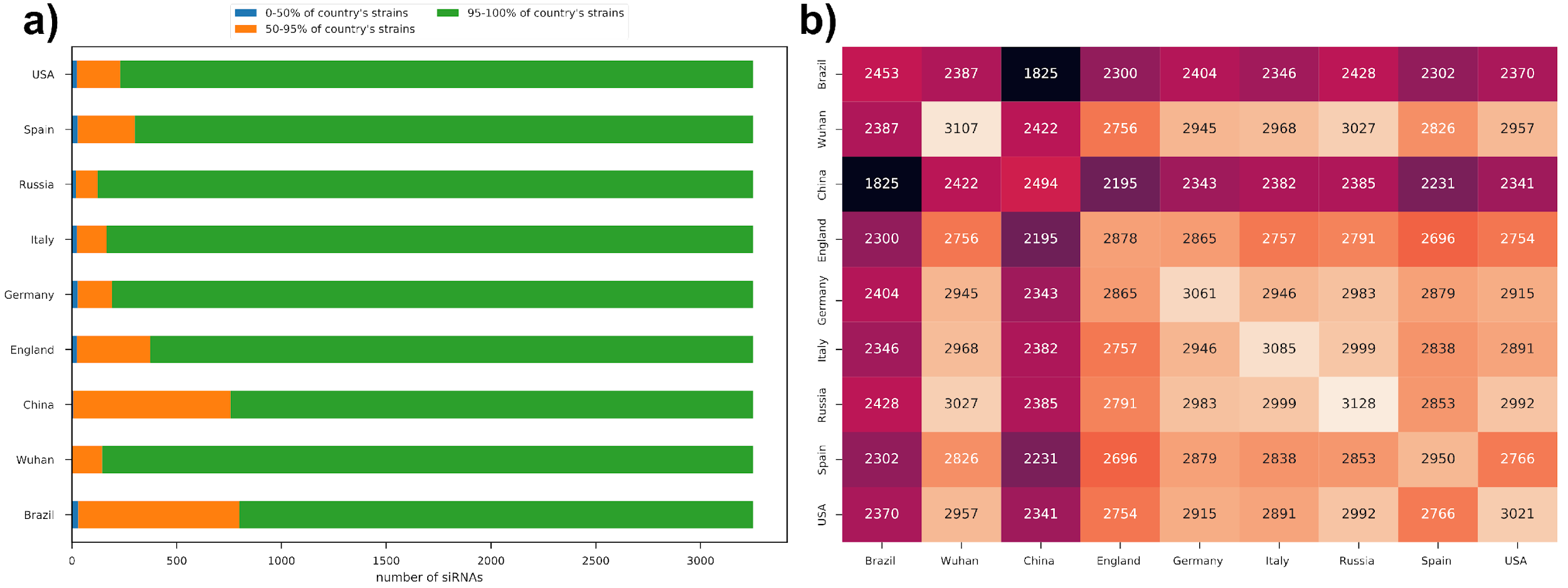
](https://paperpile.com/c/1HKrLK/s7J5+6pR6) across nine countries, divided in three layers: (i) 0-50% of the country’s strains; (ii) 50-95% of the country’s strains; (iii) 95-100% of the country’s strains; b) Intersection matrix displaying the number of siRNAs with coverage higher than 95% that each country pair shares.

**Supplementary Figure S2. Number of 21nt siRNAs considered atoxic by the literature across SARS-CoV-2 genome.**


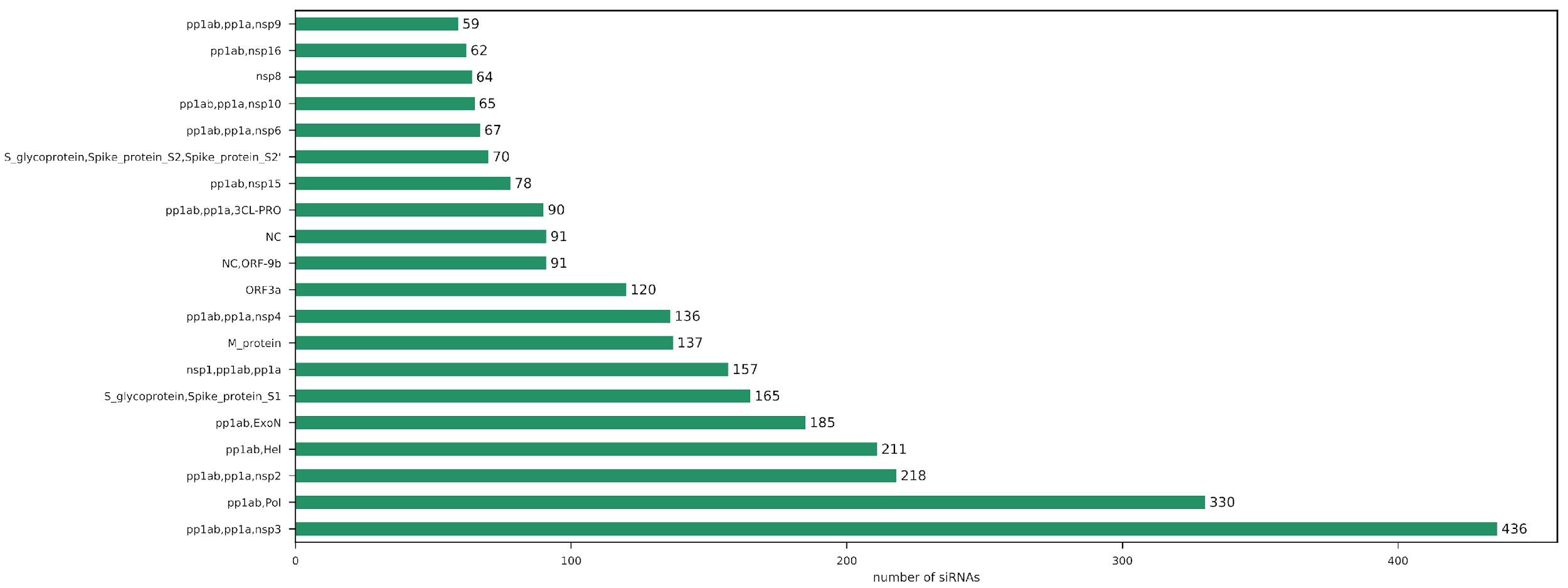
Number of 21nt siRNA targets considered atoxic per gene, displayed in horizontal bars. Overlapping genes are displayed at the same line, separated by comma.
